# Social communication and brain network properties: cognitive predictors and modulation by autistic traits

**DOI:** 10.1007/s11571-026-10481-9

**Published:** 2026-06-08

**Authors:** Elizabeth Valles-Capetillo, Diego Angeles-Valdez, Magda Giordano, Rajesh K. Kana

**Affiliations:** 1 Department of Psychology, University of Alabama at Birmingham, Birmingham, AL USA; 2 Departamento de Neurobiología Conductual y Cognitiva, Instituto de Neurobiología, Universidad Nacional Autónoma de México, Boulevard Juriquilla 3001, Querétaro, 76230 México; 3 Center for Clinical Neuroscience and Cognition, University Medical Center Groningen, University of Groningen, Groningen, The Netherlands; 4 Research School of Behavioural and Cognitive Neurosciences, University of Groningen, Groningen, The Netherlands

**Keywords:** Social communication, Cognitive resources, Autism, fMRI, Graph metrics

## Abstract

Social communication (SC) relies on the integration of linguistic processing, pragmatic inference, and theory of mind (ToM), yet its neural architecture remains insufficiently characterized. We propose and empirically test a unified SC network by integrating information neural graph theory metrics, multi-domain cognitive modeling, and autistic traits. Forty-five neurotypical adults completed a cognitive battery assessing language, executive functions, social cognition, perceptual reasoning, and the autism spectrum quotient (AQ). Global efficiency, local efficiency, and clustering coefficients were computed for language, pragmatic, and ToM networks, and their combined architecture. A replication analysis for graph-metrics and its relationship with AQ was performed using an independent sample (n = 73, 31 autistic). Results revealed that language abilities were the strongest and most consistent predictors of network efficiency, particularly within temporal-parietal nodes implicated in semantic integration and contextual interpretation. Executive functions selectively predicted efficiency within frontal control regions, while perceptual reasoning was associated with global efficiency of the precuneus, associated with social and inferential processing. Importantly, autistic traits moderated multiple brain-behavior relationships, indicating that trait-level variability shapes how cognitive abilities map onto neural efficiency within neurotypical population. The replication analysis showed partial overlap with graph-metric and AQ results. These findings advance a network-level account of SC, demonstrating that communicative competence emerges from dynamic interactions among linguistic, executive, and inferential systems, whose neural organization is tuned by individual cognitive profiles and autistic traits. This dimensional framework provides a foundation for understanding variability in social-cognitive functioning and has implications for personalized models of communication.

**Supplementary Information:**

The online version contains supplementary material available at 10.1007/s11571-026-10481-9.

## Introduction

Humans are inherently social beings, and social interactions play a critical role in mental health and psychological well-being (Holt-Lunstad et al. 2010). The ability to maintain successful social relationships is closely tied to a range of cognitive processes, including strong language skills (Rietdijk et al. 2013), and an understanding of the social use of language, i.e. pragmatics, as well as Theory of Mind (ToM) (Premack and Woodruff 1978). Language is a fundamental tool for communication, a complex system that allows people to express ideas, emotions and thoughts (Balconi 2010). However, the traditionally defined components of language are insufficient for capturing the nuances of social interaction (Martin and McDonald 2003). To navigate these complexities, it is crucial to delineate the role of communicative intentions (Bambini and Bara 2012). Pragmatics provides an interpretive framework, enabling individuals to interpret statements with multiple meanings and understand the intended message behind the words (Bosco et al. 2017). Another key cognitive process underlying communication is ToM, which refers to the ability to attribute mental states, such as beliefs and intentions, to oneself and others (Premack and Woodruff 1978).

Difficulties in ToM have been consistently reported in autistic individuals (Baron-Cohen 2000; Gao et al. 2023), along with impairments in pragmatic language processing (Ying Sng et al. 2018) and delayed language development (Mody and Belliveau 2013). These difficulties are often associated with reduced social functioning, including problems with social integration, daily living skills, employment and independent living (Bishop-Fitzpatrick et al. 2017; Gray et al. 2014). Furthermore, individuals with Autism Spectrum Disorder (ASD) often show atypical patterns across several cognitive domains, including executive functions (EF), perceptual processing, and social cognition (Magiati et al. 2014), which may further contribute to difficulties in ToM, pragmatics, and language in general (Martin and McDonald 2003; Pexman 2008; Valles-Capetillo et al. 2022; Valles-Capetillo et al. 2025a, b).

Neuroimaging techniques, such as functional magnetic resonance imaging (fMRI), have provided valuable insights into the neural basis of language, pragmatics and ToM. For instance, recent fMRI studies reveal considerable overlap in the brain regions that support these functions, including the superior temporal gyrus (STG), the middle temporal gyrus (MTG), the posterior supramarginal gyrus (pSMG), the angular gyrus (AG), the superior frontal gyrus (SFG) and the inferior frontal gyrus (IFG) (Bohrn et al. 2012; Duvall et al. 2023; Friederici 2011; Hauptman et al. 2023; Rapp et al. 2012; Reyes-Aguilar et al. 2018). Furthermore, in autistic individuals brain regions involved in ToM show reduced activation, including the anterior cingulate cortex, medial prefrontal cortex, and temporoparietal junction, these regions have been found to be involved in pragmatic language processing as well (Duvall et al. 2023; Schurz et al. 2014).

In autism, it has been showed that autistic individuals show increased IFG activation during pragmatic processing compared to the NT population, which could be a compensatory mechanism (Tesink et al. 2009). Conversely, reduced activation during pragmatic tasks has also been reported (Kotila et al. 2021), highlighting heterogeneity in neural strategies. Taken together, these findings imply that the pragmatic, language, and ToM networks overlap, and may differ in their efficiency, integration, and compensatory dynamics as a function of individual cognitive profiles and autistic traits.

Regarding the language network, a previous meta-analysis identified a distinction between local and global resting-state language connectivity (Larson et al. 2025). However, Lee et al. reported age-related changes in functional connectivity, specifically in the metric degree centrality (DC), within the language network by comparing individuals with autism spectrum disorder with typically developing participants (Lee et al. 2017). It suggests that brain regions involved in language networks should be investigated further to improve the prognosis, diagnosis and monitoring of ASD. For the ToM network, atypically increased functional connectivity was found in adolescents with ASD (Fishman et al. 2014). Likewise, no significant differences were observed between male groups with ASD and typically developing males, nor were any interactions observed between age and diagnosis with respect to global and local efficiency using functional near-infrared spectroscopy (fNIRS) (Cao et al. 2021).

Despite the recognized importance of social communication, it remains unexplored at the network-level, partly because language, pragmatics, and ToM are complex processes. Graph-theoretical approaches provide tools to quantify the efficiency, segregation, and integration of brain networks, offering insights into how information flows across systems and how neural resources are optimally, or suboptimally, allocated.

This study aims to characterize the network-level organization of the language, pragmatic, and ToM networks, conceptualized as a unified “social communication” network, in an NT sample with a wide range of autistic traits. Using graph-theoretical metrics, we examine global and local efficiency and clustering coefficients within and across these networks. We further assess how individual differences in language, EF, social cognition, and perceptual processing predict network efficiency, and whether autistic traits modulate these brain-behavior relationships. We hypothesize that (1) the language, pragmatic, and ToM networks will exhibit substantial interconnectivity, forming an integrated social communication network; (2) language abilities, EF, perceptual processing and social-cognitive functions will explain network properties; and (3) autistic-related traits will modulate the association between cognitive abilities and network properties. By integrating a dimensional approach to autistic traits with a network-based framework, this study advances our understanding of how cognitive and neural properties support social communication, providing a foundation for models of communicative variability across individuals. Furthermore, it provides a foundation for identifying neural markers relevant to variability in autistic traits.

## Method

### Participants

Forty-five Spanish-Speakers NT adults (22 female; mean age = 26.69 ± 5.83 years) participated in the study (see Table 1 and SI 1). All participants were right-handed, had no history of psychiatric or neurological disorders, which explicitly included autism spectrum disorder and attention-defficit/hyperactivity disorder. Additionally, participants provided written informed consent in accordance with the Declaration of Helsinki. Handedness was assessed using the Edinburgh Handedness Inventory (Oldfield 1971), all participants were right handed. The study was approved by the Ethics Committee of the Neurobiology Institute at the Universidad Nacional Autónoma de México (protocol #047.H.RM).

**Table 1:** Demographics characteristics and cognitive resources performance

Variable	Sample size	Variables		
	(n = 45)	Mean	SD	Range
Age (F, M)	45 (22, 23)	26.72	5.83	20–40
Education				
Science	15	27.73	6.52	18–40
Humanities	12	26.25	5.49	20–36
Engineering	18	26.11	5.66	20–40
AQ	45	15.36	7.64	2–38
Language				
Verbal fluency	45	22.87	6.94	7–39
Information	45	11.6	2.61	6–18
Similarities	45	9.84	2.53	5–17
Vocabulary	45	10.38	2.27	4–16
Executive functions				
Go-No/Go	45	309.6	10.33	227–336
Tower of London	45	8.8	2.46	2–12
Digit Span	45	7.98	2.74	3–18
N-back	45	168.96	31.49	12–218
Perceptual processing				
Block design	45	11.04	2.14	6–15
Matrix	45	10.93	2.13	4–16
Visual puzzle	45	10.82	2.38	2–15
Social cognition				
SST	45	17.07	3.92	8–26
RMET	45	25.51	4.97	9–32
IRI	45	40.2	13.83	15–92
SSS	45	3.24	1.09	1.12–5.38

*SST* short story task, *RMET* reading the mind in the eyes test, *IRI* interpersonal reactivity inventory, and *SSS* sarcasm self-report score. AQ scores above 26 suggests elevated autistic traits, above 32 points towards stronger likelihood of autism

Data used for the current project came from a broader project, results from these articles can be found in (Valles-Capetillo et al. 2025a, b; Valles-Capetillo et al. 2026). An a priori power analysis was conducted using pwr.t.test function from pwr packaged in R to determine the required sample size. Assuming a two-tailed one-sample *t*-test, an alpha level of 0.05, and a desired power of 0.80, the analysis yield a minimum sample size of 33.36 participants. To maintain adequate power while accounting for potential data loss and variability, the planned sample size was increased to 45 participants.

### Cognitive processes

To examine pragmatic ability, language, and ToM, participants completed a comprehensive neuropsychological battery. *Language abilities* were assessed using subtests from the Wechsler Adult Intelligence Scale, Fourth Edition (WAIS-IV) (Wechsler 2008), including Information, Vocabulary, and Similarities. Verbal fluency was evaluated with the Verbal Fluency test of the Batería Neuropsicológica de Funciones Ejecutivas (Flores Lázaro et al. 2008). *Social cognition* was measured using multiple instruments: the Short Story Task (SST) (Dodell-Feder et al. 2013), for affective mentalizing, the Reading the Mind in the Eyes Test (RMET) (Baron-Cohen et al. 2001a, b), for cognitive mentalizing, the Sarcasm Self-report Score (SSS) (Ivanko et al. 2004), for pragmatic inference frequency, and the Interpersonal Reactivity Inventory (IRI) (Davis 1980), for empathy. *Executive functions* were assessed via the n-back working memory task (updating), Tower of London task (ToL, planning), Go/No-go (response inhibition), and digit span (working memory), all administered using the Psychology Experiment Building Language (PEBL) software (Mueller and Piper 2014). *Perceptual reasoning* was measured using the Block Design, Visual Puzzles, and Matrix Reasoning subtests of the WAIS-IV (Wechsler 2008). *Autistic traits* were evaluated using the Autism Spectrum Quotient (AQ) (Baron-Cohen et al. 2001a, b), which measures domains including social skills, communication, attention to detail, attention switching, and imagination. Higher scores reflect greater levels of autistic traits, and scores above 26 are generally considered indicative of elevated autistic traits. Participants in the present study demonstrated a broad distribution of AQ scores ranging from 2 to 38 (see Table 1 for results). In particular, one participant scored in the range indicative of mild-to-moderate autistic traits (AQ = 26), and another scored in the range associated with a higher likelihood of autism (AQ = 38). Importantly, neither of these individuals reported a formal clinical diagnosis of autism. Additional methodological details regarding test administration are provided in Valles-Capetillo et al. (2025a, b).

### MRI acquisition and preprocessing

MRI sequences were acquired using a 3 Tesla GE MR750 scanner equipped with a 32-channel head coil. Participants were instructed to keep their eyes closed during the resting-state scan and to allow their minds to wander freely without focusing on any specific thoughts. For the anatomical images, we acquired a T1w FSPGR BRAVO sequence with the following parameters: repetition time (TR) = 0.008 s, echo time (TE) = 0.003 s, flip angle = 12° and matrix size = 256 × 256. For functional images, we used Gradient Echo Echo-Planar Imaging (GE-EPI) with TE = 0.04 s, TR = 2 s, a flip angle of 90° and a matrix size = 64 × 64. A MRIQC v.0.15 tool was used to assess the quality control of structural and functional sequences (Esteban et al. 2017). Additionally, the mean framewise displacement across the sample was below 0.5 mm, indicating the high quality of the MRI data.

Preprocessing followed standard procedures implemented in fMRIPrep (Esteban et al. 2019), including skull stripping, motion correction, slice timing correction, co-registration to anatomical images, and normalization to MNI space. Confound regressors included cerebrospinal fluid, white matter, DVARS, framewise displacement, and six motion parameters (translations and rotations).

### Selection of regions of interest

**Fig. 1:**
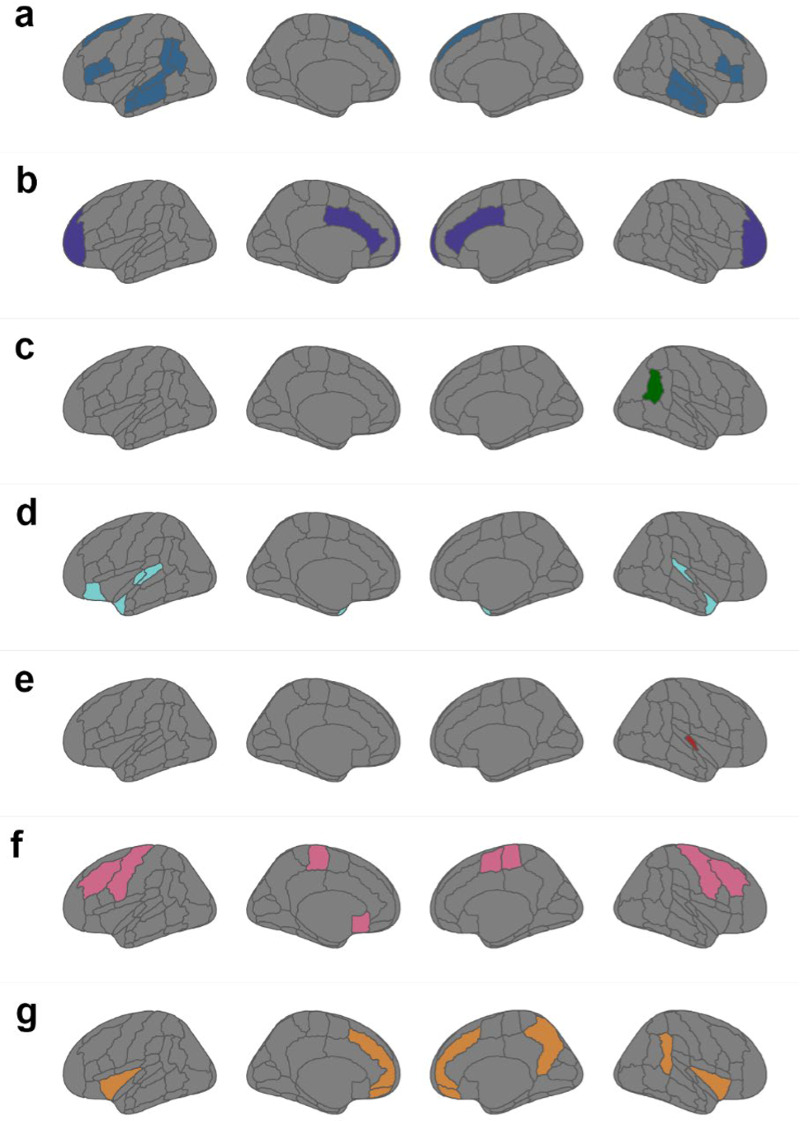
Brain regions associated with social communication. Regions were defined using the Harvard–Oxford Atlas and illustrate the spatial relationships among the pragmatic, language, and Theory of Mind (ToM) networks. Panel **a** shows the overall distribution of regions across the three networks. Panel **b** illustrates regions shared by the pragmatic and language networks. Panel **c** shows overlap between the language and ToM networks. Panel **d** shows overlap between the pragmatic and ToM networks. Panels **e**–**d** display regions uniquely associated with the pragmatic (Panel **e**), language (Panel **f**), and ToM (Panel **g**) networks, respectively

Regions of interest (ROIs) were defined based on peak coordinates from prior meta-analyses of the brain’s pragmatic (PN) (Duvall et al. 2023; Reyes-Aguilar et al. 2018), language (LN) (Hertrich et al. 2020; Lipkin et al. 2022), and ToM networks (Schurz et al. 2014). ROIs were cross-referenced with the Harvard–Oxford Atlas to ensure anatomical consistency. A total of 27 ROIs were defined for PN, 28 ROIs for LN, and 26 regions for ToM (see Fig. 1). ROI-based analyses were conducted in CONN (Whitfield-Gabrieli and Nieto-Castanon 2012) and SPM (v12.77).

### Graph theory

Fmriprep preprocessing did not include spatial smoothing, therefore functional images were smoothed using a 6-mm FWHM Gaussian kernel with the CONN toolbox (Whitfield-Gabrieli and Nieto-Castanon 2012). Additional denoising in CONN regressed out session effects and their first-order derivatives (two regressors) as well as linear trends (two regressors) for each functional run. For each participant, ROI-to-ROI correlation matrices were computed, and functional connectivity graphs were constructed by thresholding the matrices using either an absolute threshold (z > 0.5) or a relative threshold (top 10% of correlations), based on the standard procedure in the CONN Toolbox (https://web.conn-toolbox.org/fmri-methods/connectivity-measures/roi-to-roi).

Graph theory metrics were computed to characterize network properties (Achard and Bullmore 2007; Latora and Marchiori 2001), including: (i) global efficiency: average inverse shortest path length across the network, (ii) local efficiency, average efficiency of local subgraphs, and (iii) clustering coefficient, proportion of connected neighbors for each node (Fornito et al. 2016). All graph metrics were calculated separately for PN, LN, and ToM networks, and then using all the regions. Multiple comparisons were corrected using Bonferroni adjustment, with statistical significance set at *p* < 0.05.

### Multilinear regression models

To identify cognitive predictors related to the social communication network, stepwise regression models were implemented in R (R Core Team 2003) using Akaike Information Criterion (AIC) for variable selection, using the function setpAIC (both sides) from the MASS library (Bruce et al. 2020). Predictors included: language abilities (Verbal Fluency, Information, Vocabulary, Similarities), EF: (Go/No-Go, ToL, Digit Span, N-back), perceptual reasoning: (Block Design, Visual Puzzles, Matrix Reasoning) and social cognition (RMET, SST, IRI, SSS). Additionally, AQ scores were included as moderator to examine the effect of autistic traits on the relationship between graph metrics of the social communication network and cognitive predictors. To control for multiple comparisons, significance thresholds were adjusted by graph measure and cognitive domain, resulting in a corrected α of *p* < 0.001 (0.05/42). Sex, age, and profession did not show significant differences and therefore were not included as covariables (see SI 1–4).

To assess the robustness of the results, a bootstrapping procedure was implemented. This method relies on repeated resampling with replacement to approximate the sampling distribution of the estimates and evaluate their variability across resampled datasets (citation). Bootstrapped coefficients were computed using the *bootstrap_parameters* function from the *parameters* package (citation), based on 10,000 iterations and 95% confidence intervals. The relative contribution of the overall model and individual predictors was further quantified using the *calc.relimp* function (type = “lmg”) from the *relaimpo* package. The LMG metric decomposes the model’s total explained variance (*R*^2^) by averaging each predictor’s contribution across all possible orderings of entry into the model, thereby providing a robust estimate of each variable’s independent contribution while accounting for shared variance among predictors. Furthermore, regression model assumptions were examined using the *performance* package (citation), including tests of normality, heteroscedasticity, and the identification of influential outliers.

Outliers were removed prior to analysis. Outlier detection was performed to minimize bias associated with extreme values. Analyses were conducted in R using the rstatix package (Kassambara 2023). Extreme outliers were defined as observations exceeding the third quartile (Q3) + 3 × interquartile range (IQR) or falling below the first quartile (Q1) − 3 × IQR. Outlier removal was conducted at the variable level; thus, participants were excluded only from specific analyses in which outliers were detected and retained in analyses for domains/ROIs without outliers (see SI 5 for full details).

### Replication analysis

Participants from autism brain imaging data exchange (ABIDE) I and II were included in the replication analysis. Those sites with AQ were selected, in total, three sites were included (ABIDE I: Leuven Sample 1; ABIDE II: IU and SBL). Across the three sites, 99 subjects were included. Participants were excluded if they don’t have AQ scores (n = 3); problems in the preprocessing (n = 1, reaction time from json did not match with the nifti), were left handed (n = 8) or Ambi handed (n = 5); their age did not match with the young adult sample included (adolescent = 1, middle age = 8). The final sample was 73 participants, 31 autistic (1 F, 30 M, age: mean = 23.8, range = 19–37) and 42 non-autistic (4 F, 38 M, age: mean = 25.95, range = 18–39).

Quality assessment and preprocessing of the data were the same as describe in “MRI acquisition and preprocessing” and “Graph theory” sections. The mean FD was 0.1 (0.03–0.41) In addition to the previous described methods, two additional covariables were included in the graph theory analysis, site and sex. This is to control differences by the three different sites included and sex regarding the discrepancy in sex ratio (1:4).

To examine the relationship between graph-theoretical metrics and AQ scores, we conducted mixed-effects regression analyses using the *lme4* package in R. Consistent with “Multilinear regression models” section, graph metrics computed at both the whole-network level and at the level of individual nodes were entered as dependent variables, with AQ included as the primary predictor. Sex was included as a fixed-effect covariate to account for the disproportionate sex distribution in the sample, and site was modeled as a random effect to control for between-site variability. In contrast to the models described in “Multilinear regression models” section, cognitive measures were not included in these analyses. Similar as “Multilinear regression models” section, bootstraps were performed across significant models. This analysis was adopted to specifically evaluate the robustness and consistency of the association between AQ scores and graph-theoretical properties of the social communication network.

## Results

### Graph-metrics

#### Network effects

Across the combined set of nodes from the LN, PN, and ToM networks, graph metrics revealed statistically significant effects (*p* < 0.000001) in Global Efficiency (*β* = 0.44, *T*(44) = 71.33), Local Efficiency (*β* = 0.63, *T*(44) = 70.26), and Clustering Coefficient (*β* = 0.51, *T*(44) = 54.49). When the individual networks were assessed, global efficiency was higher in language (0.44 ± 0.04), followed by PN (0.38 ± 0.06) and ToM (*β* = 0.37 ± 0.09), with significant differences between them (*χ*^*2*^(2) = 41.86, *p* < 0.0.001), in particular between LN with PN (*p* < 0.001) and ToM (*p* < 0.001; SI 6A). In local efficiency, the LN showed the highest local efficiency (0.64 ± 0.08), followed by ToM (0.62 ± 0.12), and PN (0.59 ± 0.16), but the differences were not statistically significant (*χ*^*2*^(2) = 4.94, *p* = 0.08; SI 6b). Finally, mean clustering coefficients were again highest in the ToM network (0.55 ± 0.13), followed by LN (0.51 ± 0.08) and PN (0.50 ± 0.15), with no significant group differences (*χ*^*2*^(2) = 4.30, *p* = 0.12; SI 6C). Given the few differences between networks, the following section focuses on the joint contributions of the three networks. Results for each individual region are presented separately in SI 7.

#### Individual-nodes effects

**Fig. 2:**
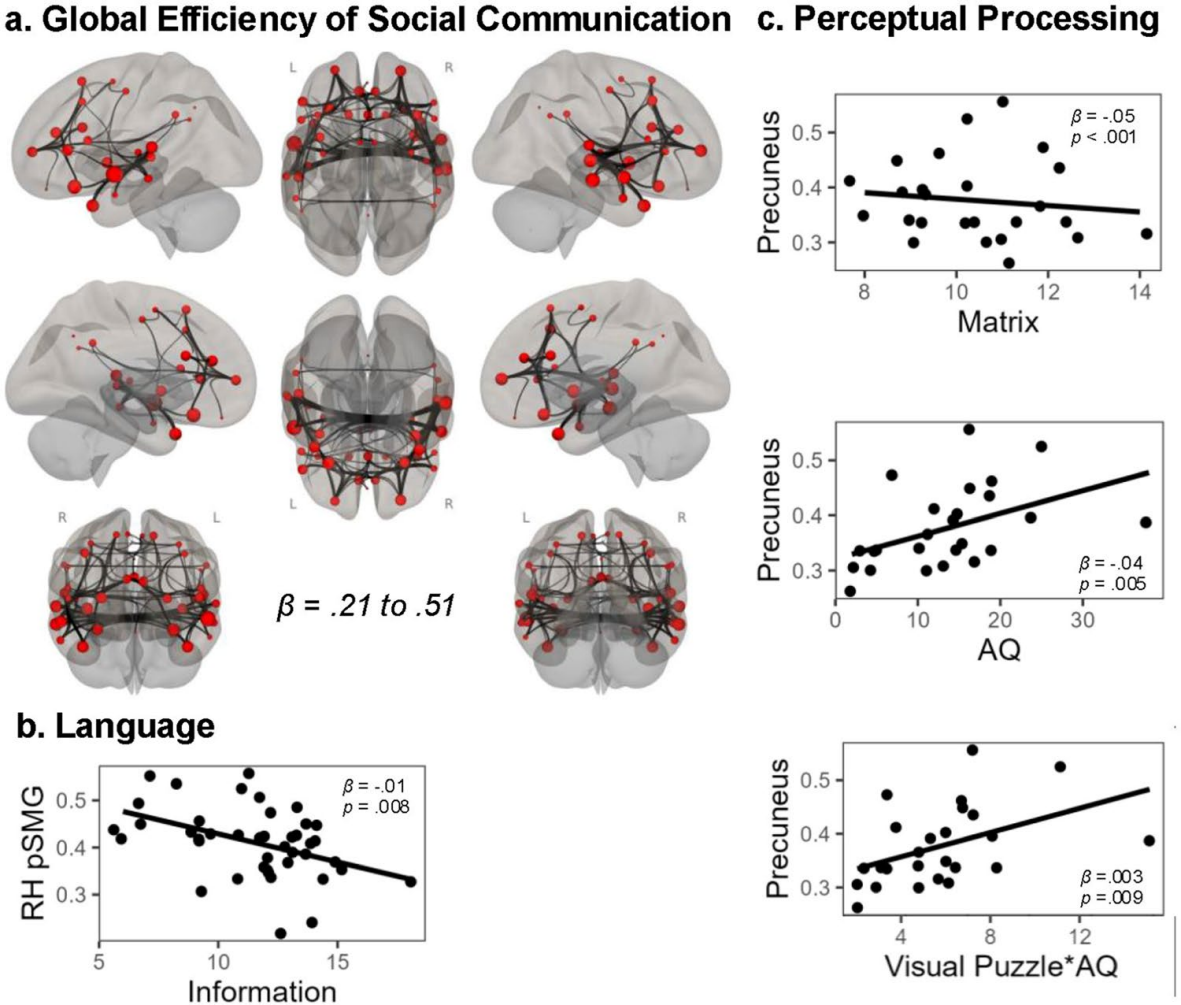
Global efficiency of the social communication network. Panel a shows the beta coefficients for global efficiency. Panel b illustrates the association between right pSMG and language abilities, whereas Panel c shows the corresponding relationship between precuneus and perceptual processing. Beta coefficients reflect results from the multilinear regression analyses. *RH* right hemisphere, *pSMG* posterior supramarginal gyrus, *AQ* autism spectrum quotient. An asterisk (*) indicates a moderated relationship between variables

Global efficiency showed significant associations across nearly all ROIs. The strongest effects were observed bilaterally (left *β* = 0.48, right *β* = 0.49) in the right posterior MTG (pMTG, *β* = 0.50), and right posterior STG (pSTG, *β* = 0.51). Additional high beta values were found in the frontal pole, temporal pole, insula, and planum temporale (*β* = 0.44 to 0.49). Moderate associations were found in the Heschl’s gyrus, precentral gyrus, and SMG (*β* = 0.38 to 0.43) (Fig. 2a).

**Fig. 3:**
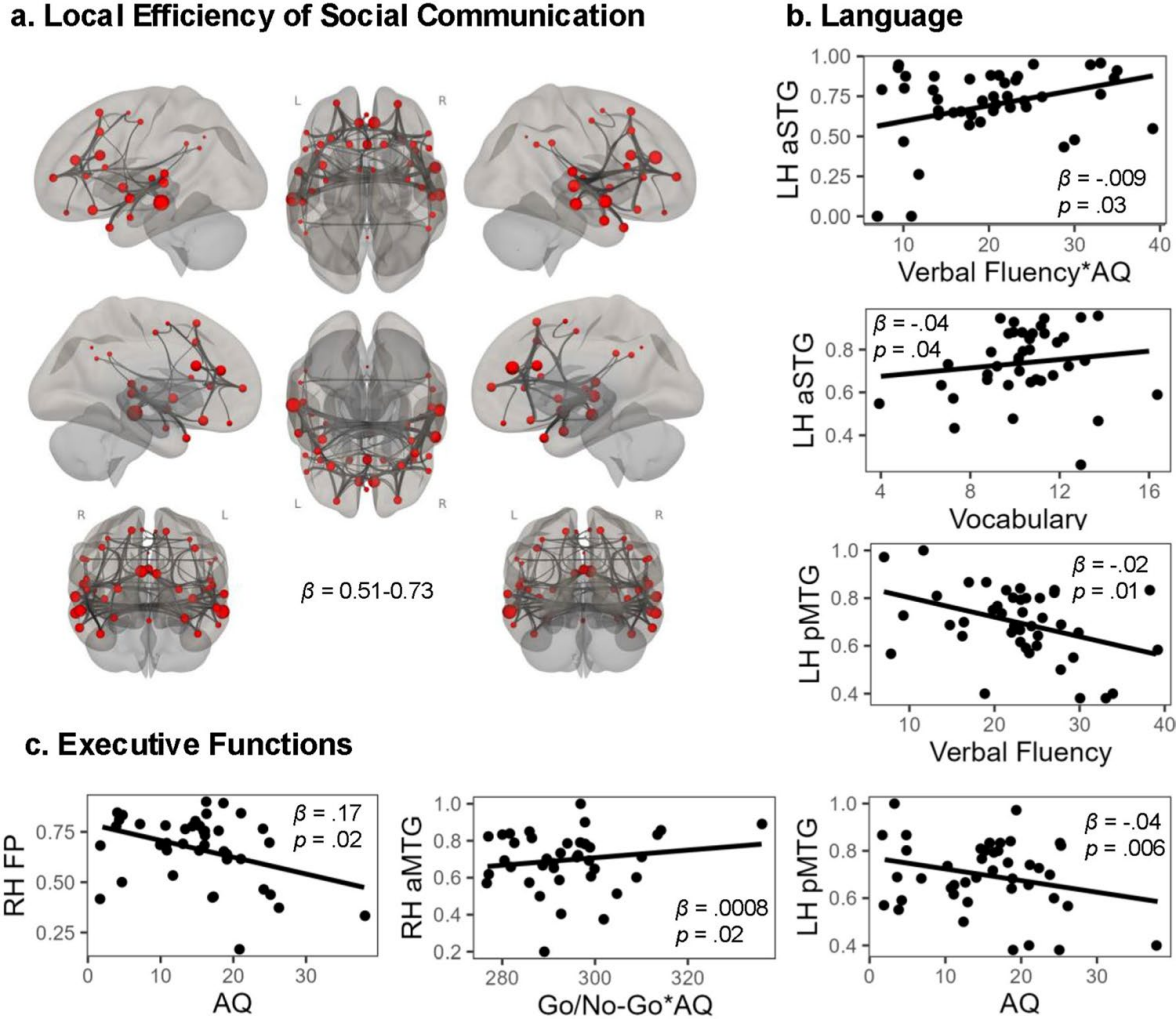
Local efficiency of the social communication network. Panel a shows the beta coefficients for local efficiency of the multilinear regression models. Panel b illustrates the association between left anterior superior temporal gyrus and language abilities, whereas Panel c shows the relationship between left posterior middle temporal gyrus and perceptual processing. Beta coefficients reflect results from the multilinear regression analyses. *RH* right hemisphere, *pSMG* posterior supramarginal gyrus, *AQ* autism spectrum quotient. An asterisk (*) indicates a moderated relationship between variables

Local efficiency yielded the largest overall effect sizes. Peak associations were found in right anterior STG (aSTG, *β* = 0.73), medial frontal cortex (MedFC; *β* = 0.73), and bilaterally in the pMTG (left *β* = 0.70, right *β* = 0.70). Strong effects were also observed in right pSTG (*β* = 0.70), anterior cingulate (*β* = 0.72), and bilaterally in the paracingulate gyrus, insular cortex, and temporal pole regions (*β* range: 0.63 to 0.66) (Fig. 3a).

**Fig. 4:**
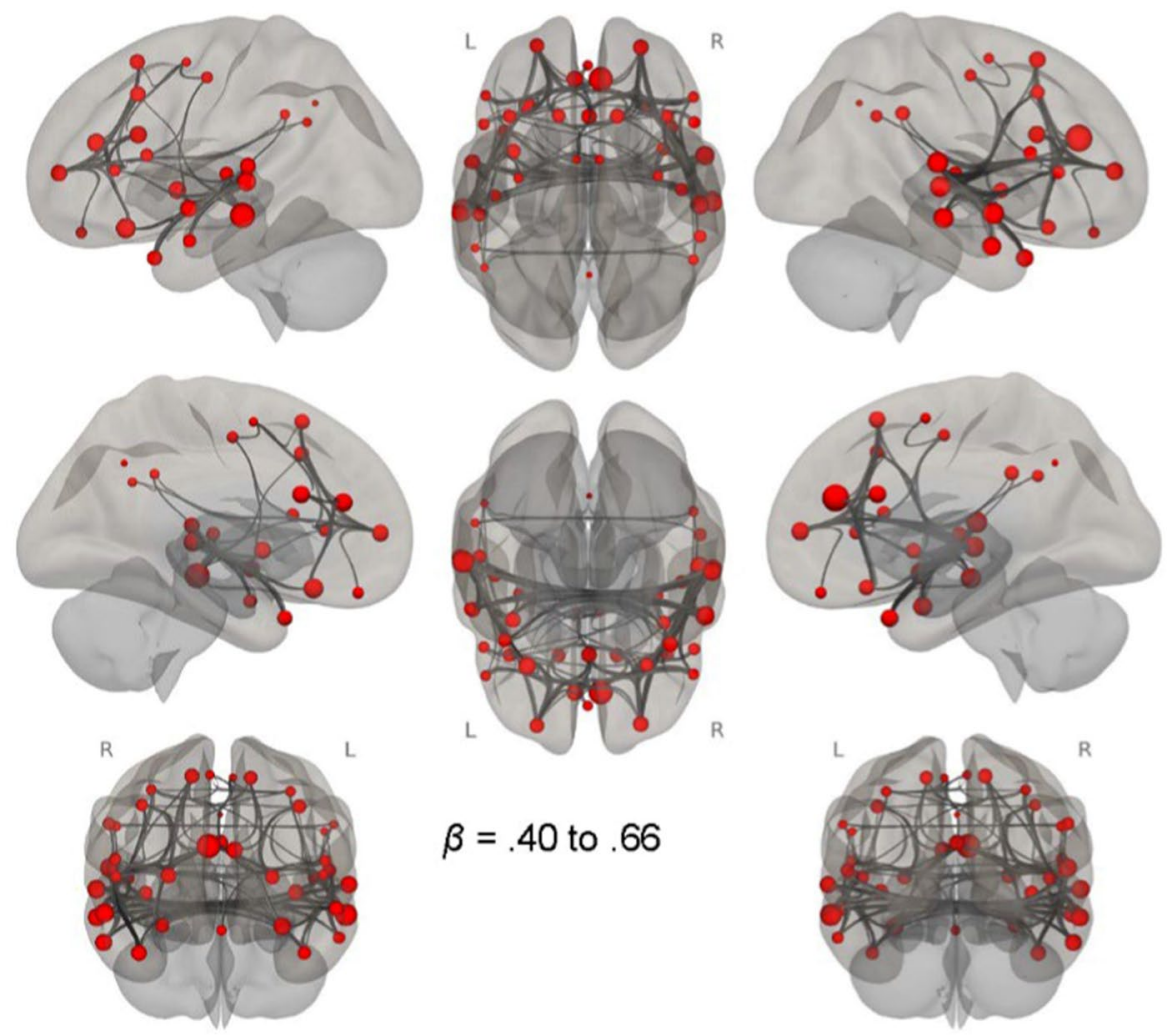
Clustering coefficient of the social communication network

Clustering coefficients mirrored the pattern of local efficiency. Strongest effects emerged in right aSTG (*β* = 0.57), right pSTG (*β* = 0.54), right pMTG (*β* = 0.53), and anterior cingulate (*β* = 0.57). Additional associations were found in orbitofrontal cortex, frontal pole, IFG (*β* range: 0.45 to 0.52), planum temporale, and Heschl’s gyrus (*β* = 0.50 to 0.58). Medial frontal cortex (*β* = 0.66) and precuneus (*β* = 0.60) also contributed significantly (Fig. 4).

### Cognitive predictors of graph-theoretical metrics

Details from the significant regression models can be found in SI 8.

#### Global efficiency

Cognitive predictors of global efficiency varied by node. In the right pSMG, *language abilities* significantly predicted global efficiency (*F*(6, 36) = 4.69, *p* < 0.001, adj *R*^*2*^ = 0.34), accounting for 43.88% of the variance explained by the model. Higher Information scores were associated with lower global efficiency in the right pSMG (*β* = − 0.01, *p* = 0.008), accounting for 15.0% of the explained variance (see Fig. 2b).

In contrast, global efficiency in the precuneus was explained by *perceptual processing* (*F*(4,40) = 5.86, *p* = 0.0008, adj *R*^*2*^ = 0.30), accounting for 36.91% of the variance. Higher Matrix Reasoning scores were associated with lower precuneus global efficiency (*β* = − 0.05, *p* < 0.001), while higher AQ scores were also related to reduced global efficiency in this node (*β* = − 0.04, *p* = 0.005), accounting for 17.55% and 2.56% of the explained variance, respectively. In addition, a significant interaction between AQ and Visual Puzzle was associated with higher precuneus global efficiency (*β* = 0.003, *p* = 0.009), explaining 6.26% of the model’s variance (see Fig. 2c).

#### Local efficiency

*Language abilities* significantly predicted local efficiency in temporal areas. In the left aSTG, the overall model was significant (*F*(6,37) = 4.69, *p* < 0.001, adj. *R*^*2*^ = 0.35), with language accounting for 43.98% or the model’s variance. Verbal fluency (*β* = 0.04, *p* = 0.02), vocabulary (*β* = 0.04, *p* = 0.04), and the AQ × verbal fluency interaction (*β* = − 0.009, *p* = 0.03) emerged as significant predictors, explaining 8.80%, 10.58% and 12.11% of the model’s variance, respectively (see Fig. 3b). Similarly, in the left pMTG the model was significant (*F*(5,37) = 5.28, *p* < 0.001, adj. *R*^*2*^ = 0.33), with language measures explaining 41.67% of the variance. Significant independent predictors included verbal fluency (*β* = − 0.02, *p* = 0.012) and AQ (*β* = − 0.04, *p* = 0.006), accounting for 16.41% and 6.81%, respectively (see Fig. 3c).

*Executive functions* significantly predicted local efficiency in right frontal pole (*F*(5,37) = 5.55, *p* < 0.001, adj. *R*^*2*^ = 0.35), explaining 42.87% or the variance. The AQ (*β* = 0.17, *p* = 0.02) was the primary contributor, accounting for 9.51% of the model’s variance. The local efficiency in the right aMTG was also significant (*F*(5,33) = 3.64, *p* = 0.009, adj. *R*^*2*^ = 0.26), explaining 35.57% of the variance. The AQ (*β* = − 0.27, *p* < 0.0*5*) and the interaction between AQ and Go/No-Go (*β* = 0.0008, *p* < 0.0*5*) were the significant predictors, explaining 13.71% and 10.79%, respectively, of the model’s variance (see Fig. 3d).

#### Clustering coefficient

Although the regression models were significant at an uncorrected threshold, none survived Bonferroni correction for multiple comparisons.

### Replication analysis

#### Comparison between autistic and non-autistic samples

No significant differences were found between groups in global efficiency in LN (*T*(69) = − 0.80,* β* = − 0.01, *p-uncorrected* = 0.42), PN (*T*(69) = − 1.87,* β* = − 0.02, *p-uncorrected* = 0.06), ToM (*T*(69) = − 0.36,* β* = 0.00, *p-uncorrected* = 0.72), and all combined (SC, *T*(69) = − 1.32,* β* = − 0.01, *p-uncorrected* = 0.19). Local efficiency did not showed significant differences between groups in LN (*T*(69) = − 2.70,* β* = − 0.04, *p-uncorrected* = 0.008), PN (*T*(69) = 0.53,* β* = 0.01, *p-uncorrected* = 0.59), ToM (*T*(69) = − 0.31,* β* = − 0.01, *p-uncorrected* = 0.75), and all combined (SC, *T*(69) = − 0.84,* β* = − 0.01, *p-uncorrected* = 0.40). Finally, clustering efficiency did not showed significant between group differences in LN (*T*(69) = − 1.92,* β* = − 0.03, *p-uncorrected* = 0.06), PN (*T*(69) = 0.46,* β* = 0.01, *p-uncorrected* = 0.65), ToM (*T*(69) = − 0.52,* β* = − 0.01, *p-uncorrected* = 0.60), and all combined (SC, *T*(69) = − 0.68,* β* = − 0.01, *p-uncorrected* = 0.50). At the nodal level, the number of significant nodes and associated degrees of freedom varied across networks, reflecting heterogeneity in node-wise effects across participants, particularly within the autistic sample. Full betas and degrees of freedom for each network and sample (combined, autism and neurotypical) can be found in SI 9–11.

#### Graph metrics and AQ relationship

**Fig. 5:**
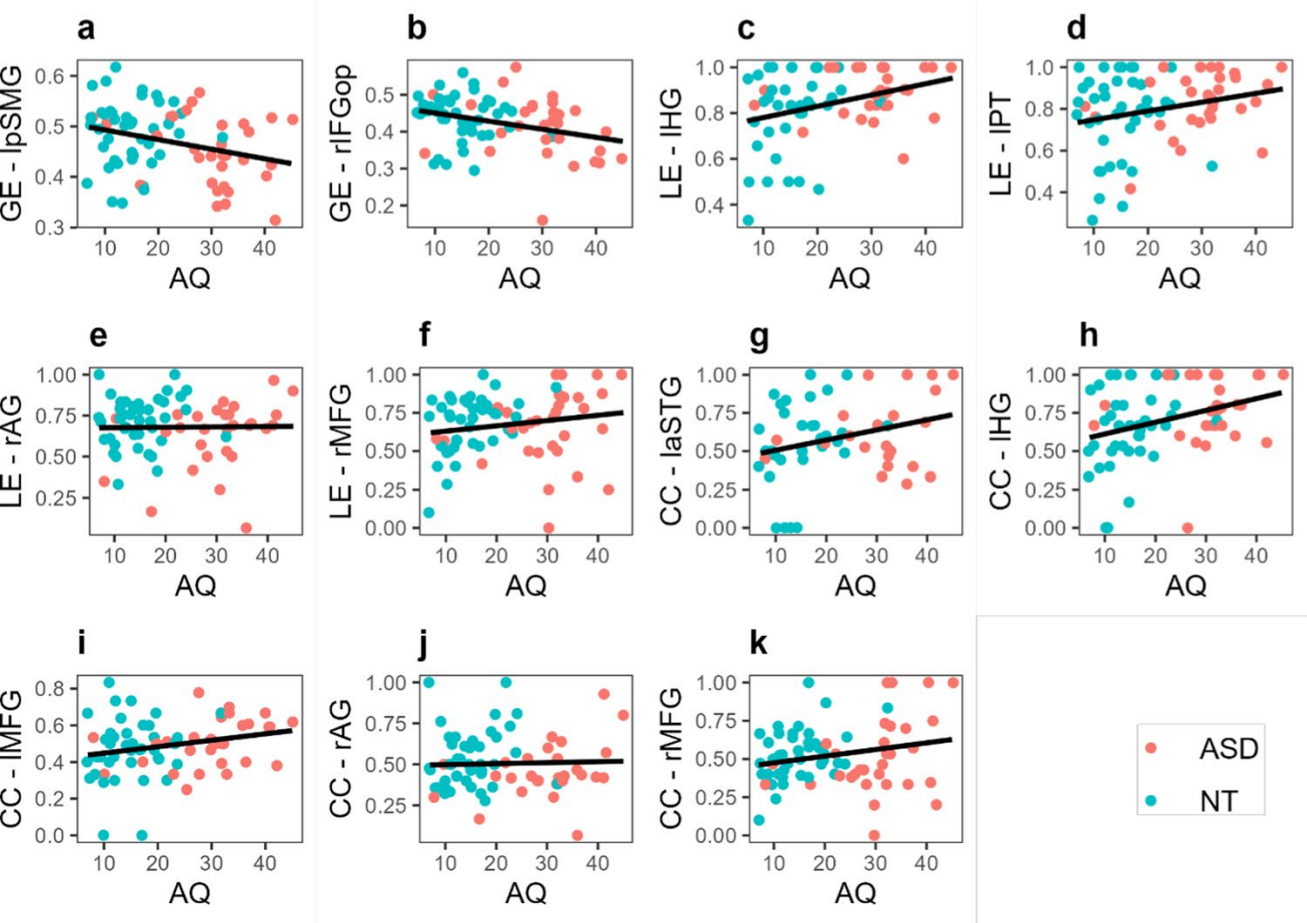
Associations between autistic traits and graph-theoretical metrics. Significant node-level effects are displayed. Panels **a**, **b** illustrate global efficiency in the left posterior supramarginal gyrus (**a**) and right inferior frontal gyrus pars opercularis (**b**). Panels **c**–**f** show local efficiency in the left Heschl’s gyrus (**c**), left planum temporale (**d**), right angular gyrus (**e**), right middle frontal gyrus (**f**). Panels **g**–**k** show clustering coefficient in the left anterior superior temporal gyrus (**g**), left middle frontal gyrus (**h**), left Heschel gyrus (**i**), right angular gyrus (**j**), right middle frontal gyrus (**k**). Only statistically significant models are presented

Mixed-effects regression analyses revealed significant associations between graph metrics and autistic traits. Specifically, lower AQ scores were associated with reduced global efficiency in the left pSMG (*β* = − 1.94, *p* = 0.01) and right IFG opercularis (*β* = − 2.34, *p* = 0.03). In contrast, higher AQ scores were positively associated with local efficiency in the left Heschl’s gyrus (*β* = 6.93, *p* = 0.01) and planum temporale (*β* = 5.35, *p* = 0.02). Similarly, clustering coefficient showed positive associations with AQ scores in the left aSTG (*β* = 6.55, *p* = 0.041), left Heschel gyrus (*β* = 7.37, *p* = 0.008) and left MFG (*β* = 3.57, *p* = 0.039, see Fig. 5). Full regression model results are reported in the SI 12.

## Discussion

**Fig. 6:**
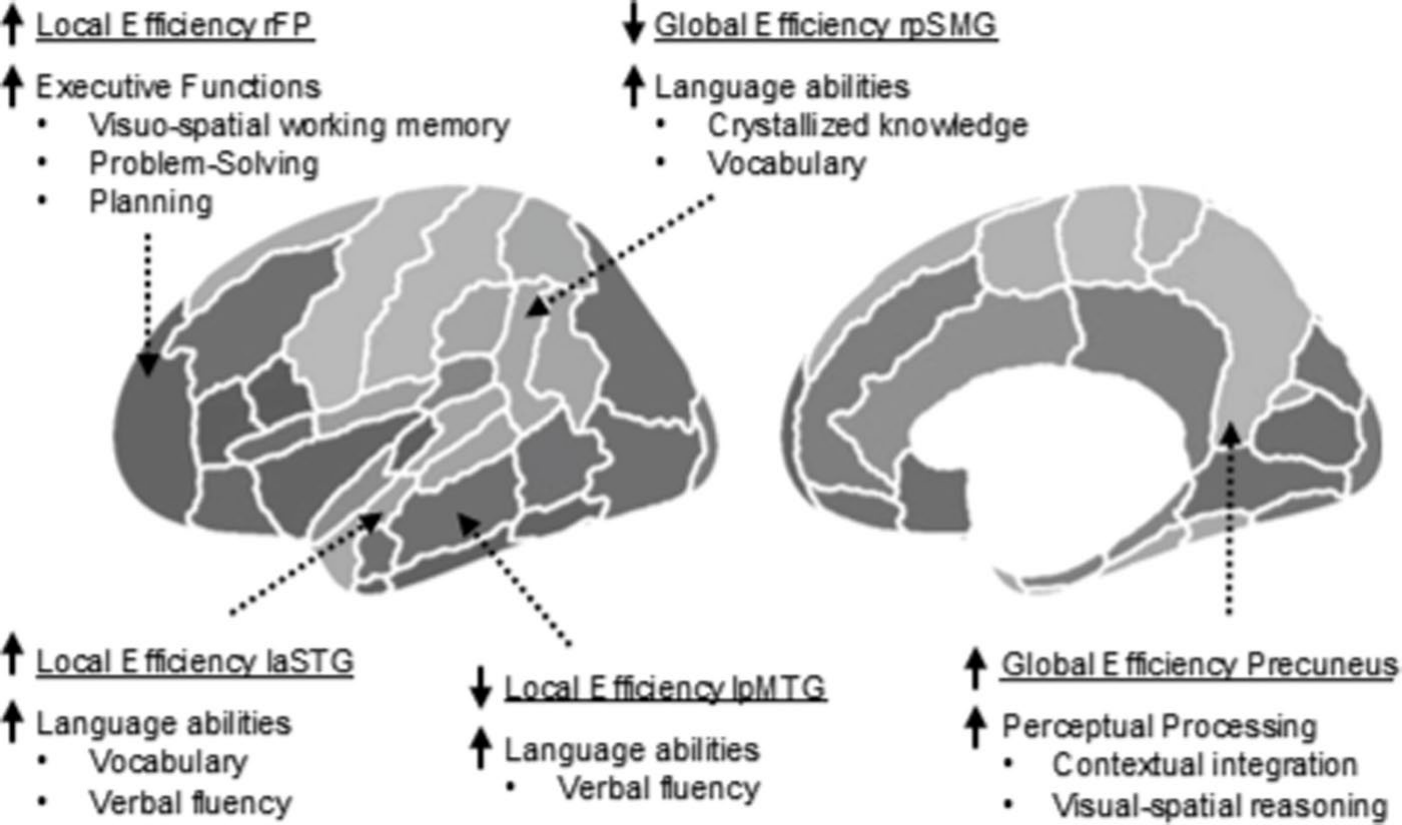
Schematic illustration of the relationships between graph-theoretical metrics and cognitive resources at the node level. Arrows show the direction of associations (positive or negative). *rFP* right frontal pole, *rpSMG* right posterior supramarginal gyrus, *laSTG* left anterior superior temporal gyrus, *lpMTG* left posterior middle temporal gyrus

This study investigated the network-level organization of pragmatic, language, and ToM networks and examined how individual differences in cognitive resources relate to this organization and how this relationship is modulated by autistic traits. Three principal findings emerged. First, pragmatic, language, and ToM regions formed a highly overlapping and efficiently organized network, supporting a unified architecture for social communication. Second, language abilities, EF, and perceptual reasoning differentially predicted network efficiency across key hubs, indicating domain-specific contributions within a shared system (see Fig. 6). Third, autistic traits systematically moderated multiple brain–behavior relationships, demonstrating that trait-level variability reshapes how cognitive abilities map onto neural efficiency even within the NT range. Finally, analyses in an independent sample from the ABIDE dataset revealed no group-level differences in network organization in pragmatic, language, ToM and social communication networks, and showed relationship with autistic traits. Together, these findings advance a dimensional, neural network-based account of social communication and help reconcile variability in findings across prior neuroimaging studies.

### Functional organization of social communication network

Consistent with meta-analytic evidence, we found substantial overlap among the pragmatic, language, and ToM networks, with regions spanning temporal, frontal, and parietal contributing to global and local efficiency as well as clustering (Duvall et al. 2023; Hauptman et al. 2023; Reyes-Aguilar et al. 2018). Core nodes included bilateral anterior MTG, pMTG, IFG (opercularis, triangularis and orbitalis), SFG, and left pSTG and AG, regions known to support linguistic integration, semantic access, and social inference (Friederici 2011; Tikochinski et al. 2025). These results reinforce the notion that social communication underlies neurocognitive systems supporting semantic control and integration (Binney and Ramsey 2020).

When the three systems were modeled together as a unified *social communication network*, all regions contributed to efficiency and clustering metrics, underscoring a distributed architecture optimized for flexible communication. These result suggests a shared efficiency backbone that supports flexible transitions between linguistic decoding, pragmatic inference, and mentalizing (Hauptman et al. 2023; Thye et al. 2024; Valles-Capetillo et al. 2025a, b). Collectively, these findings support a network-based account of social communication in which linguistic and social-cognitive processes are intrinsically interdependent, providing a mechanistic framework for understanding individual differences and vulnerability across communicative contexts. This convergence aligns with network-level perspectives on brain organization, whereby cost-efficient topologies simultaneously support functional specialization and large-scale integration (Achard and Bullmore 2007; Fornito et al. 2016; Latora and Marchiori 2001; Wang et al. 2011).

### Language as core predictor of network efficiency

Across domains, language abilities emerged as the most consistent predictor of network efficiency within the social communication system. Notably, strongly crystallized language knowledge, associated with lower global efficiency in the right pSMG, was associated with stronger linguistic abilities, particularly broader crystallized knowledge indexed by Information scores, and word knowledge and semantic knowledge assessed by vocabulary. The pSMG is a key parietal hub supporting lexical-semantic integration, phonological processing, and perspective-taking, supporting the integration of linguistic meaning with contextual and social information (Spotorno et al. 2012; Thye et al. 2023; Zhang et al. 2020). This inverse relationship is consistent with the neural efficiency hypothesis, whereby greater expertise is associated with more selective, less diffuse network engagement (Di Domenico et al. 2015; Prat et al. 2007). Higher crystallized knowledge may reduce the need for extensive pSMG mediated communication, reflecting more automatized, resource-efficient processing within the social communication network. Prior work has similarly shown that increased language proficiency is accompanied by reduced functional connectivity in this region (Valles-Capetilloet al. 2025a). Together, these findings suggest that crystallized language and semantic knowledge expertise supports social communication by enabling more streamlined network configurations, with pSMG contributions selectively recruited only under heightened contextual or perspective-taking demands.

Language abilities also predicted local efficiency in the left temporal area, particularly in aSTG and pMTG. These regions have been classically associated with language processing (Friederici 2011) and have been implicated in pragmatic language (Reyes-Aguilar et al. 2018) and ToM (Schurz et al. 2014) processing. Higher local efficiency in the aSTG was associated with stronger vocabulary and verbal fluency, consistent with evidence from transcranial magnetic stimulation (TMS) demonstrating a causal role of the left aSTG in speech perception (Ramos Nuñez et al. 2020). This region is further modulated by semantic content and linguistic complexity, showing preferential responses to social–emotional concepts and increasing activation with larger linguistic units (Mellem et al. 2016). In contrast, lower local efficiency in the pSMG was associated with stronger verbal fluency, suggesting that reduced segregation in this region may facilitate broader network integration. Consistent with this interpretation, altered functional connectivity of this region has been linked to social communication difficulties (Yun et al. 2017), and has been proposed to mediate the integration of automatic semantic retrieval with goal-directed cognition (Davey et al. 2016). Its involvement in both verbal and non-verbal semantic processing further underscores its role as a multimodal semantic hub (Hoffman et al. 2012). These findings suggest that language proficiency is supported by complementary efficiency profiles within left temporal regions, with increased local specialization in aSTG and more integrative, less segregated processing in pMTG jointly facilitating fluent and flexible language use.

Taken together, these findings indicate that superior language performance is supported not by uniformly increased connectivity, but by region-specific and computationally dependent optimization within the social communication network. Linguistic expertise appears to promote efficient, automatized processing characterized by reduced global integration in some nodes alongside enhanced local specialization in others.

### Executive function contributions to frontal connectivity

EF predicted local efficiency in the right frontal pole. The frontal pole is implicated in higher-order executive control, cognitive flexibility, and adaptive behavior, and structural properties of this region have been linked to social functioning and problem-solving abilities (Levan et al. 2016). Lesion studies further suggest that the frontal pole supports disengagement from ongoing cognitive sets and reallocation of resources toward novel goals (Mansouri et al. 2015). Increased local efficiency in this region may therefore facilitate adaptive executive control during complex communicative contexts, where planning and flexibility are required to navigate social demands.

### Perceptual processing and global efficiency via the precuneus

Perceptual reasoning abilities, particularly Matrix Reasoning and Visual Puzzle performance, predicted global efficiency in the precuneus, a region consistently implicated in social communication, contextual integration, and inferential processing (Desai et al. 2011; Schmidt and Seger 2009; Shibata et al. 2011; Valles-Capetillo et al. 2025a, b; van Ackeren et al. 2012; Yang et al. 2016). Matrix reasoning reflects pattern detection, relational reasoning, and inference generation. On the other hand, Visual puzzle assess visual-spatial reasoning and the ability to analyze abstract visual information (Wechsler 2008), cognitive operations central to social communication, which requires integrating linguistic input with contextual cues and representations of other’s mental states (Grau-Husarikova et al. 2024; Hadad and Segal 2025; Maquate and Knoeferle 2021). This interpretation suggests that nonliteral and socially nuanced communicative forms preferentially recruit neural mechanisms that support contextual integration and inferential processing beyond core linguistic decoding (Feng et al. 2017; Hauptman et al. 2023; Hertrich et al. 2020; Reyes-Aguilar et al. 2018). This is also consistent with task-based evidence showing that communicative forms with high inferential demands, such as irony, robustly recruit the precuneus (Valles-Capetillo et al. 2025a, b). Together, these findings suggest that perceptual reasoning abilities support social communication by optimizing global integration within the precuneus, facilitating efficient coordination of inferential, contextual, and mental state representations.

### Autistic traits as a modulator of brain-behavior relationships

A central contribution of this study is the demonstration that autistic traits systematically moderate brain-behavior relationships across multiple cognitive domains and network nodes. AQ scores interacted with language, executive, and perceptual predictors in regions including pSMG, precuneus, aSTG, pMTG, and frontal pole, indicating that trait-level variation reshapes the neural correlates of cognition even within an NT population. Importantly, this pattern was partially replicated in an independent sample including both neurotypical and autistic individuals, where the pSMG and aSTG remained significant predictors of social communication network organization. The replication sample also revealed additional associations involving global efficiency in the right IFGop, as well as segregation measures in the left Heschel gyrus and planum temporale and the right AG and MFG. These effects suggest partial convergence with the primary findings while also extending the observed pattern to broader language-related and frontoparietal systems, likely reflecting differences in sample composition. These findings support a dimensional model of social-cognitive variability, consistent with the evidence that autistic traits capture meaningful individual differences beyond categorical diagnoses (Baron-Cohen et al. 2001a, b). Rather than acting as additive risk factors, autistic traits appeared to reorganize the mapping between cognitive abilities and neural efficiency, suggesting reliance on distinct strategies or neural pathways. This perspective challenges binary notions of neurotypicality and neurodivergence and instead emphasizes individualized neural architectures shaped by continuous variation in cognitive style.

### Theoretical and translational implications

Collectively, these results support a network-level account in which social communication emerges from coordinated interactions among language, executive, perceptual, and social-cognitive systems. The consistent modulation by autistic traits underscores the necessity of accounting for individual differences when modeling the neural bases of communication. From a translational standpoint, these findings suggest that interventions aimed at improving social communication, particularly in neurotypical population and potentially neurodivergent populations, may benefit from targeting not only core linguistic skills but also executive and inferential processes, tailored to individual trait profiles. However, experimental studies that directly manipulate these cognitive domains and assess their impact on social communication outcomes are needed to establish causal pathways and inform intervention design.

### Limitations

An important limitation is the sample size, while typical for resting-state fMRI studies with intensive cognitive characterization, limits statistical power and generalizability, especially for looking at individual variability. Replication in larger samples will be important to confirm the robustness of the observed brain–behavior relationships and moderation effects. In particular, the inclusion of more diverse samples, such as participants spanning a broader age range and varied sociocultural backgrounds, would enhance the ability to capture developmental trajectories and individual differences. Secondly, the exclusive use of a neurotypical adult sample, in the cognitive resources analyses, precludes direct inferences about autistic populations. Although the dimensional AQ framework provides valuable insights into trait-related variability, future studies should extend this approach to clinically diagnosed autistic adults to evaluate whether similar network principles apply. Finally, the reliance on resting-state fMRI limits conclusions about task-specific neural dynamics. This limitation is particularly relevant given that the current findings revealed higher global efficiency in the language network compared with pragmatic and ToM networks. Resting-state connectivity captures intrinsic network organization but may not fully reflect the context-dependent recruitment of social communication systems observed during active language, pragmatic or mentalizing tasks. Combining resting-state and task-based approaches will be critical for linking intrinsic network properties to communicative performance.

## Conclusion

Together, these findings advance our understanding of the neural architecture supporting social communication by demonstrating that language, executive function, and perceptual reasoning are embedded within an efficient and dynamic network system shaped by individual cognitive profiles and autistic traits. The integration of language, pragmatic, and Theory of Mind networks into a unified social communication framework reflects their joint contribution to encoding linguistic content, contextual interpretation, and mental state inference, processes that are inherently interdependent in naturalistic communication. While analyses of individual networks provide functional specificity, examining them as a combined system offers a complementary perspective by capturing cross-network interactions and emergent organizational properties that may not be apparent in isolation. Consistent with this view, the findings support a dimensional model of social-cognitive functioning, in which trait-level variation systematically modulates the relationship between behavior and brain network organization. By revealing how these interactions unfold across the pragmatic, language, and Theory of Mind networks, this work lays the groundwork for more personalized models of assessment and intervention. Future research should extend this framework to clinical and developmental populations, where the potential to enhance communicative functioning through targeted modulation of neural efficiency is both a scientific and translational priority.

## Supplementary Information

Below is the link to the electronic supplementary material.

Supplementary file1 (DOCX 1315 KB)

## Data Availability

The data files generated and analyzed in this study are publicly available from the OpenNEURO database (10.18112/openneuro.ds004533.v1.0.0).

## Code availability

Code and the data required to reproduce the analyses reported in this study are openly available on GitHub git@github.com:Eli1404/Social-Communication.git.
