# Supplementary material for: Social communication and brain network properties: cognitive predictors and modulation by autistic traits
Source: Cogn Neurodyn. 2026 Jun 8;20(1):115. doi: 10.1007/s11571-026-10481-9 (PMC13243155; doi:10.1007/s11571-026-10481-9)
Supplement: Supplementary file 1 — Supplementary file1 (DOCX 1315 KB) [file 11571_2026_10481_MOESM1_ESM.docx]

**Social Communication and Brain Network Properties: Cognitive Predictors and Modulation by Autistic Traits**

Elizabeth Valles-Capetillo^1,2*+^, Diego Angeles-Valdez^2,3,4+^, Magda Giordano^2^, Rajesh K. Kana^1^,

^1^Department of Psychology, University of Alabama at Birmingham, Birmingham, AL, USA

^2^Departamento de Neurobiología Conductual y Cognitiva, Instituto de Neurobiología, Universidad Nacional Autónoma de México, Boulevard Juriquilla 3001, Querétaro 76230, México

^3^Cognitive Neuroscience Center, Department of Biomedical Sciences, University Medical Center Groningen, University of Groningen, The Netherlands.

^4^Research School of Behavioural and Cognitive Neurosciences, University of Groningen, Groningen, The Netherlands.

+ Contributed equally to this work.

**Corresponding author:** ecapetil@uab.edu

**Journal:** Cognitive Neurodynamics

**Supplementary Information**

**SI 1.** Demographics characteristics and cognitive resources performance by Sex.

| **Variable** | **Female**  **(n = 22)** | | | **Male**  **(n = 22)** | | | **Comparison** |
| --- | --- | --- | --- | --- | --- | --- | --- |
|  | *Mean* | *SD* | *Range* | *Mean* | *SD* | *Range* | *P-Value* |
| Age (F, M) | 26.73 | 6.65 | 22-40 | 26.65 | 5 | 18-37 | 0.97 |
| AQ | 14.32 | 8.45 | 2-38 | 16.35 | 6.81 | 2-26 | 0.69 |
| Language | | | | | | | |
| Verbal Fluency | 23.41 | 5.96 | 12-39 | 22.35 | 7.87 | 7-38 | 0.77 |
| Information | 11.91 | 2.63 | 6-18 | 11.3 | 2.6 | 6-15 | 0.71 |
| Similarities | 10.18 | 2.61 | 5-17 | 9.52 | 2.47 | 5-15 | 0.69 |
| Vocabulary | 10.73 | 2.07 | 7-16 | 10.04 | 2.44 | 4-14 | 0.69 |
| Executive Functions | | | | | | | |
| Go-No/Go | 309.09 | 12.97 | 277-336 | 310.09 | 7.24 | 290-320 | 0.8 |
| Tower of London | 8.27 | 2.6 | 2-12 | 9.3 | 2.24 | 4-12 | 0.65 |
| Digit Span | 7.46 | 2.87 | 3-18 | 8.48 | 2.57 | 4-13 | 0.69 |
| N-back | 166.23 | 24.5 | 64-190 | 171.56 | 37.36 | 12-218 | 0.77 |
| Perceptual Processing | | | | | | | |
| Block Design | 10.91 | 2.33 | 7-15 | 11.17 | 1.99 | 6-14 | 0.78 |
| Matrix | 10.77 | 1.88 | 8-14 | 11.09 | 2.37 | 4-16 | 0.77 |
| Visual Puzzle | 11.54 | 1.95 | 7-15 | 10.13 | 2.58 | 2-14 | 0.65 |
| Social Cognition | | | | | | | |
| SST | 16.91 | 3.9 | 11-26 | 17.22 | 4.02 | 8-25 | 0.8 |
| RMET | 26.64 | 3.61 | 18-32 | 24.43 | 5.88 | 9-31 | 0.65 |
| IRI | 43.73 | 11.32 | 16-65 | 36.83 | 15.36 | 15-92 | 0.65 |
| SSS | 49.73 | 11.32 | 16-65 | 36.83 | 15.36 | 15-92 | 0.69 |
| **Abbreviations:** SST = Short Story Task, RMET = Reading the Mind in the Eyes Test, IRI = Interpersonal Reactivity Inventory, and SSS = Sarcasm Self-report Score.  **Note:** AQ scores above 26 suggest elevated autistic traits, above 32 points towards stronger likelihood of autism. | | | | | | | |

**SI 2. Comparison of Autism Spectrum Quotient by Sex.**


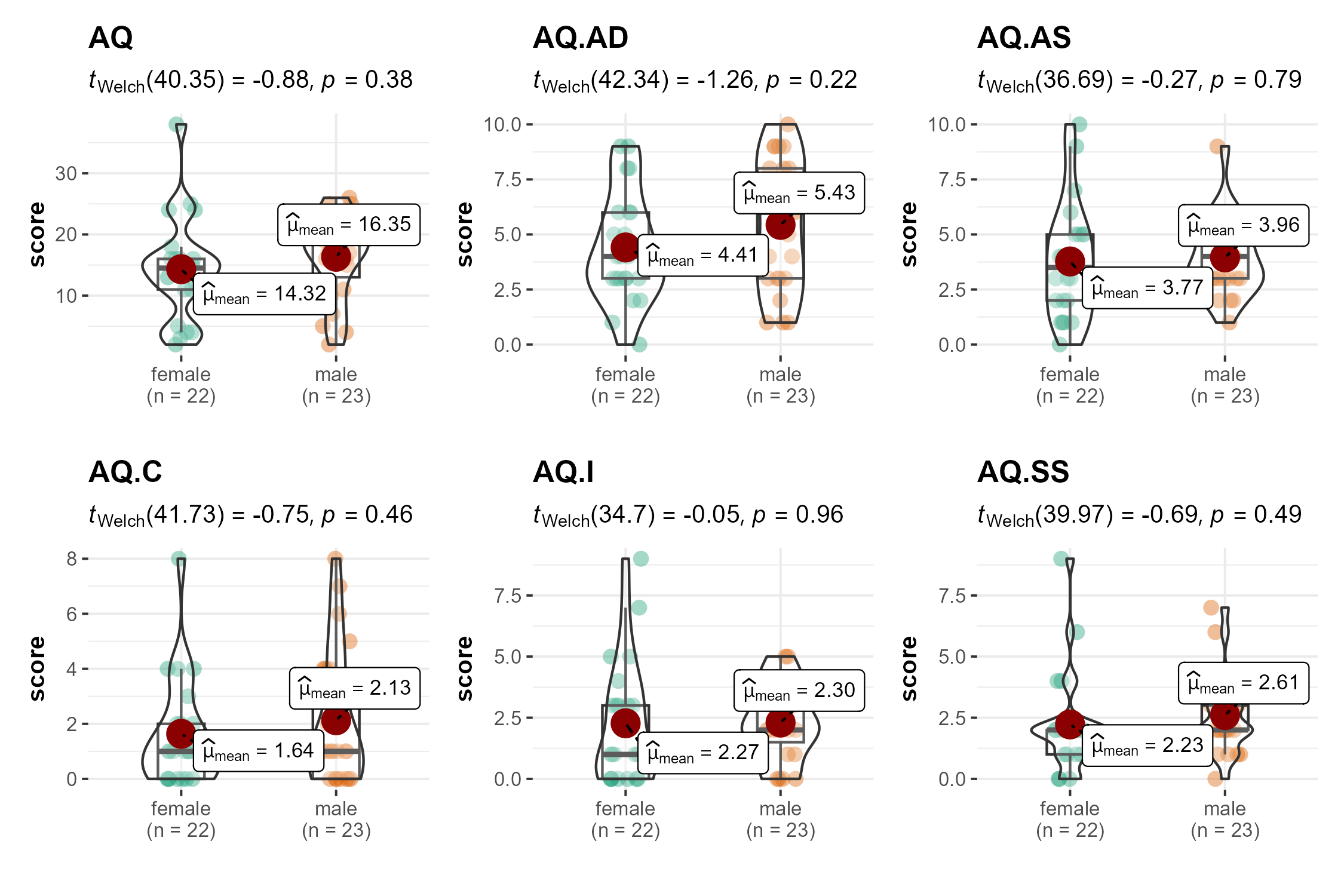
Abbreviations: AQ = Autism spectrum Quotient, AD = attention detail, AS = attention switching, C = communication, I = Imagination, SS = social skills.

**SI 3. Comparison of Autism Spectrum Quotient by Profession.**


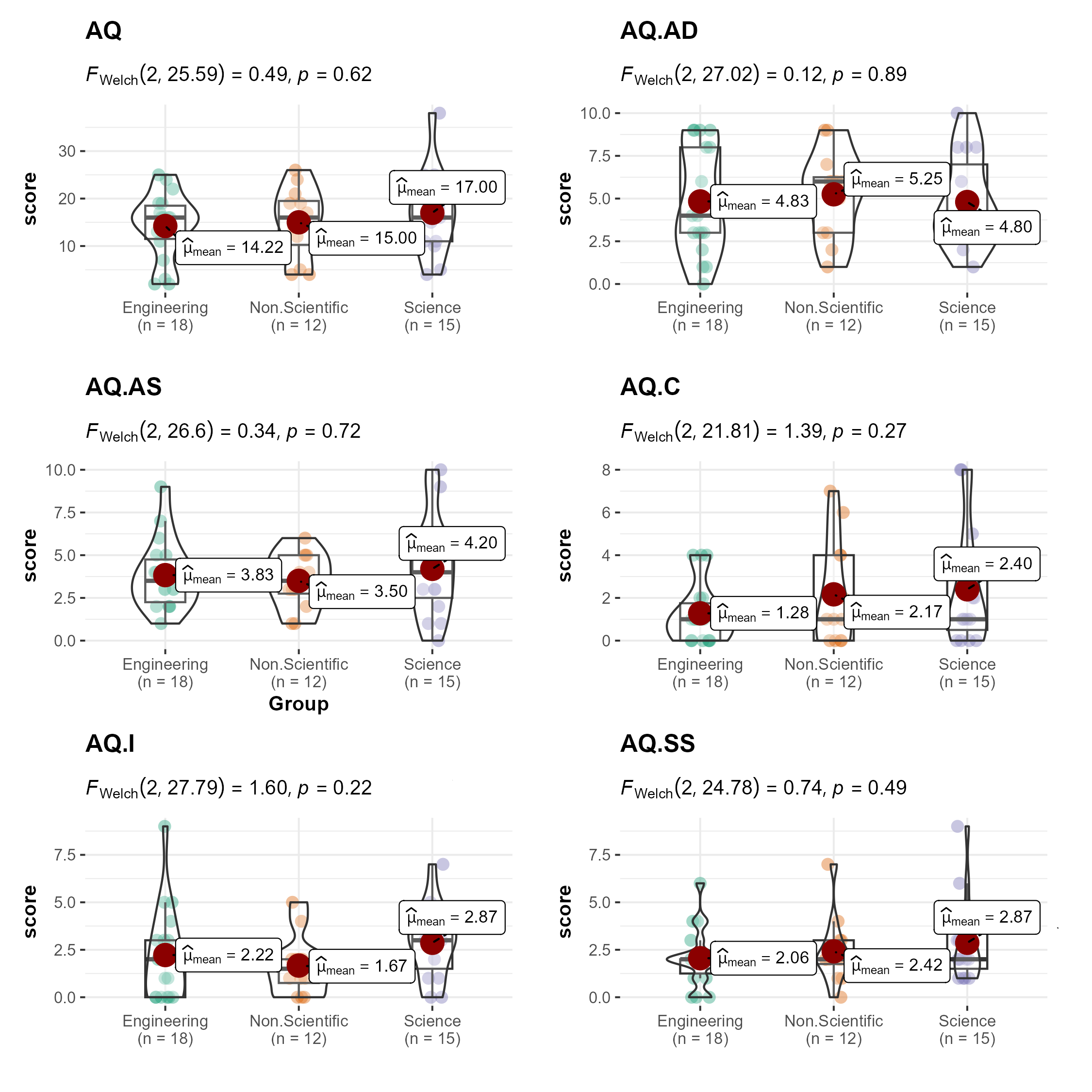
Abbreviations: AQ = Autism spectrum Quotient, AD = attention detail, AS = attention switching, C = communication, I = Imagination, SS = social skills.

**SI 4. Correlation of Autism Spectrum Quotient and Age.**


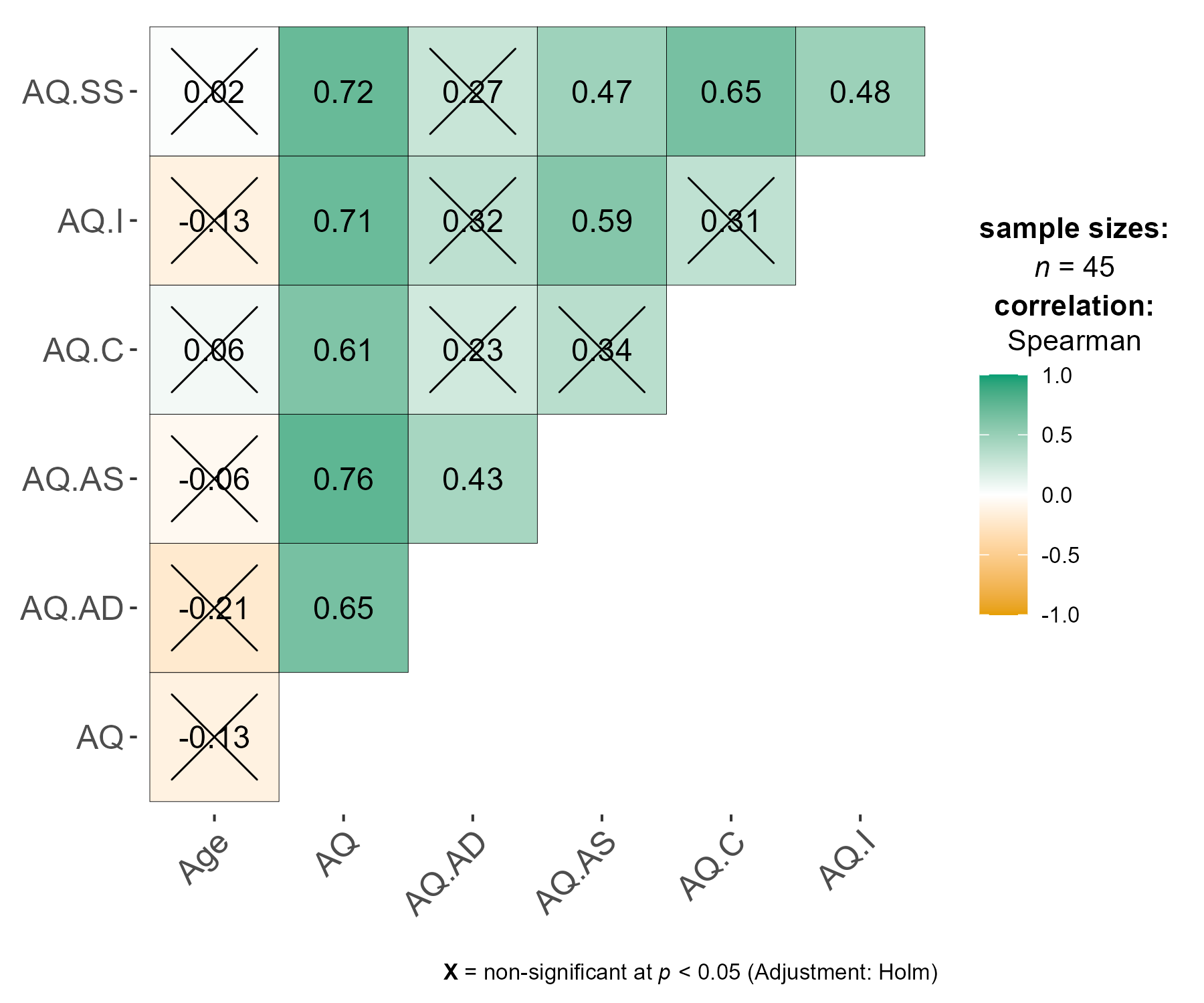


Abbreviations: AQ = Autism spectrum Quotient, AD = attention detail, AS = attention switching, C = communication, I = Imagination, SS = social skills.

**SI 5.** Outliers identified and excluded across cognitive domains and graph-theoretical measures.

| **Participant** | **Variable** | **Network** | **ROI** | **Age** | **Sex** | **Group** |
| --- | --- | --- | --- | --- | --- | --- |
| 6038 | IRI | NA | NA | 31 | male | Engineering |
| 6035 | Nback | NA | NA | 26 | male | Engineering |
| 6041 | Nback | NA | NA | 21 | female | Non.Scientific |
| 6036 | Global Efficiency | Language | All | 36 | male | Engineering |
| 6036 | Global Efficiency | Language | LH.FP | 36 | male | Engineering |
| 6030 | Global Efficiency | Language | LH.HG | 27 | male | Engineering |
| 6055 | Global Efficiency | Language | LH.HG | 21 | male | Engineering |
| 6027 | Global Efficiency | Language | LH.IFGop | 25 | male | Science |
| 6027 | Global Efficiency | Language | LH.MFG | 25 | male | Science |
| 6036 | Global Efficiency | Language | LH.MFG | 36 | male | Engineering |
| 6036 | Global Efficiency | Language | LH.PaCiG | 36 | male | Engineering |
| 6036 | Global Efficiency | Language | LH.SFG | 36 | male | Engineering |
| 6020 | Global Efficiency | Language | LH.aMTG | 36 | male | Non.Scientific |
| 6027 | Global Efficiency | Language | LH.aMTG | 25 | male | Science |
| 6036 | Global Efficiency | Language | LH.aMTG | 36 | male | Engineering |
| 6036 | Global Efficiency | Language | LH.pMTG | 36 | male | Engineering |
| 6055 | Global Efficiency | Language | LH.pMTG | 21 | male | Engineering |
| 6036 | Global Efficiency | Language | LH.pSMG | 36 | male | Engineering |
| 6038 | Global Efficiency | Language | LH.pSTG | 31 | male | Engineering |
| 6028 | Global Efficiency | Language | RH.HG | 24 | female | Engineering |
| 6029 | Global Efficiency | Language | RH.HG | 28 | female | Non.Scientific |
| 6048 | Global Efficiency | Language | RH.IFGtr | 37 | male | Science |
| 6056 | Global Efficiency | Language | RH.IFGtr | 35 | female | Non.Scientific |
| 6030 | Global Efficiency | Language | RH.PaCiG | 27 | male | Engineering |
| 6026 | Global Efficiency | Language | RH.PreCG | 21 | male | Science |
| 6062 | Global Efficiency | Language | RH.PreCG | 26 | male | Engineering |
| 6055 | Global Efficiency | Language | RH.aMTG | 21 | male | Engineering |
| 6055 | Global Efficiency | Language | RH.pMTG | 21 | male | Engineering |
| 6026 | Global Efficiency | Language | RH.pSMG | 21 | male | Science |
| 6059 | Global Efficiency | Language | RH.pSMG | 23 | female | Science |
| 6056 | Global Efficiency | Pragmatic | LH.aSTG | 35 | female | Non.Scientific |
| 6036 | Global Efficiency | Pragmatic | LH.pMTG | 36 | male | Engineering |
| 6055 | Global Efficiency | Pragmatic | LH.pMTG | 21 | male | Engineering |
| 6038 | Global Efficiency | Pragmatic | LH.pSTG | 31 | male | Engineering |
| 6018 | Global Efficiency | Pragmatic | RH.aMTG | 18 | male | Science |
| 6055 | Global Efficiency | Pragmatic | RH.aMTG | 21 | male | Engineering |
| 6036 | Global Efficiency | SC | All | 36 | male | Engineering |
| 6036 | Global Efficiency | SC | LH.FP | 36 | male | Engineering |
| 6030 | Global Efficiency | SC | LH.HG | 27 | male | Engineering |
| 6055 | Global Efficiency | SC | LH.HG | 21 | male | Engineering |
| 6027 | Global Efficiency | SC | LH.IFGop | 25 | male | Science |
| 6027 | Global Efficiency | SC | LH.MFG | 25 | male | Science |
| 6036 | Global Efficiency | SC | LH.MFG | 36 | male | Engineering |
| 6036 | Global Efficiency | SC | LH.PaCiG | 36 | male | Engineering |
| 6036 | Global Efficiency | SC | LH.SFG | 36 | male | Engineering |
| 6020 | Global Efficiency | SC | LH.aMTG | 36 | male | Non.Scientific |
| 6027 | Global Efficiency | SC | LH.aMTG | 25 | male | Science |
| 6036 | Global Efficiency | SC | LH.aMTG | 36 | male | Engineering |
| 6036 | Global Efficiency | SC | LH.pMTG | 36 | male | Engineering |
| 6055 | Global Efficiency | SC | LH.pMTG | 21 | male | Engineering |
| 6036 | Global Efficiency | SC | LH.pSMG | 36 | male | Engineering |
| 6038 | Global Efficiency | SC | LH.pSTG | 31 | male | Engineering |
| 6028 | Global Efficiency | SC | RH.HG | 24 | female | Engineering |
| 6029 | Global Efficiency | SC | RH.HG | 28 | female | Non.Scientific |
| 6048 | Global Efficiency | SC | RH.IFGtr | 37 | male | Science |
| 6056 | Global Efficiency | SC | RH.IFGtr | 35 | female | Non.Scientific |
| 6030 | Global Efficiency | SC | RH.PaCiG | 27 | male | Engineering |
| 6026 | Global Efficiency | SC | RH.PreCG | 21 | male | Science |
| 6062 | Global Efficiency | SC | RH.PreCG | 26 | male | Engineering |
| 6055 | Global Efficiency | SC | RH.aMTG | 21 | male | Engineering |
| 6055 | Global Efficiency | SC | RH.pMTG | 21 | male | Engineering |
| 6026 | Global Efficiency | SC | RH.pSMG | 21 | male | Science |
| 6059 | Global Efficiency | SC | RH.pSMG | 23 | female | Science |
| 6013 | Global Efficiency | ToM | LH.IFGtr | 26 | male | Science |
| 6048 | Global Efficiency | ToM | LH.IFGtr | 37 | male | Science |
| 6030 | Global Efficiency | ToM | LH.PaCiG | 27 | male | Engineering |
| 6036 | Global Efficiency | ToM | LH.PaCiG | 36 | male | Engineering |
| 6044 | Global Efficiency | ToM | LH.PaCiG | 21 | female | Engineering |
| 6055 | Global Efficiency | ToM | LH.pMTG | 21 | male | Engineering |
| 6055 | Global Efficiency | ToM | RH.aMTG | 21 | male | Engineering |
| 6015 | Local Efficiency | Language | AC | 30 | female | Science |
| 6034 | Local Efficiency | Language | AC | 30 | male | Non.Scientific |
| 6016 | Local Efficiency | Language | RH.aMTG | 26 | male | Engineering |
| 6018 | Local Efficiency | Language | RH.aMTG | 18 | male | Science |
| 6039 | Local Efficiency | Language | RH.aSTG | 29 | male | Engineering |
| 6015 | Local Efficiency | SC | AC | 30 | female | Science |
| 6034 | Local Efficiency | SC | AC | 30 | male | Non.Scientific |
| 6016 | Local Efficiency | SC | RH.aMTG | 26 | male | Engineering |
| 6018 | Local Efficiency | SC | RH.aMTG | 18 | male | Science |
| 6039 | Local Efficiency | SC | RH.aSTG | 29 | male | Engineering |
| 6037 | Local Efficiency | ToM | MedFC | 29 | male | Engineering |
| 6038 | Local Efficiency | ToM | MedFC | 31 | male | Engineering |
| 6034 | Local Efficiency | ToM | RH.aSTG | 30 | male | Non.Scientific |
| 6060 | Local Efficiency | ToM | RH.aSTG | 22 | male | Non.Scientific |
| 6061 | Local Efficiency | ToM | RH.aSTG | 20 | female | Engineering |

**SI 6.** Comparisons of graph metrics across brain networks. Panels show group-level comparisons between the language network (LN), pragmatic network (PN), and Theory of Mind (ToM) network for (A) global efficiency, (B) local efficiency, and (C) clustering coefficient. Each dot represents an individual participant; higher values indicate greater values.


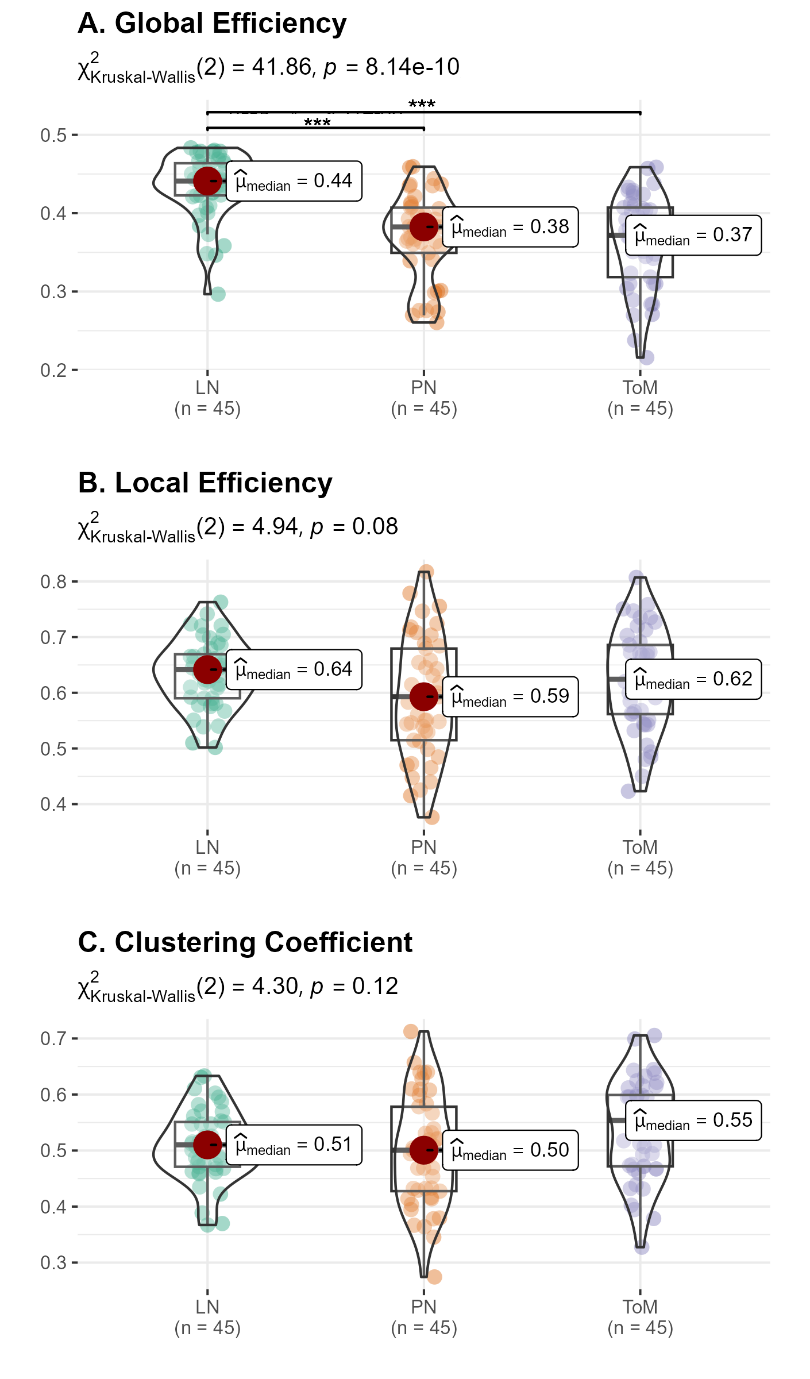


**SI 7.-** Betas per graph metrics and network.

| **Lobule** | **ROI** | **Global Efficiency** | | | | **Local Efficiency** | | | | **Clustering Coefficient** | | | |
| --- | --- | --- | --- | --- | --- | --- | --- | --- | --- | --- | --- | --- | --- |
|  |  | **SC** | **LN** | **PN** | **ToM** | **SC** | **LN** | **PN** | **ToM** | **SC** | **LN** | **PN** | **ToM** |
| Frontal | IFGor l | 0.48 | 0.39 | 0.38 | n.s. | 0.65 | 0.55 | 0.49 | n.s | 0.49 | 0.46 | 0.42 | n.s |
| Frontal | IFGr r | 0.47 | 0.39 | 0.38 | n.s. | 0.65 | 0.5 | 0.58 | n.s | 0.52 | 0.43 | 0.49 | n.s |
| Frontal | FP l | 0.46 | n.s | 0.36 | 0.42 | 0.56 | n.s | 0.44 | 0.54 | 0.43 | n.s | 0.39 | 0.45 |
| Frontal | FP r | 0.49 | n.s | 0.37 | 0.42 | 0.62 | n.s | 0.55 | 0.62 | 0.45 | n.s | 0.49 | 0.53 |
| Frontal | IFGop l | 0.42 | 0.37 | 0.27 | 0.32 | 0.57 | 0.48 | 0.4 | 0.41 | 0.48 | 0.43 | 0.38 | 0.38 |
| Frontal | IFG op r | 0.4 | 0.35 | 0.26 | 0.29 | 0.66 | 0.61 | 0.35 | 0.43 | 0.55 | 0.54 | 0.3 | 0.39 |
| Frontal | IFGtr l | 0.44 | 0.39 | 0.34 | 0.38 | 0.58 | 0.49 | 0.5 | 0.53 | 0.5 | 0.44 | 0.45 | 0.47 |
| Frontal | IFGtr r | 0.45 | 0.39 | 0.36 | 0.37 | 0.61 | 0.48 | 0.46 | 0.6 | 0.49 | 0.38 | 0.4 | 0.54 |
| Frontal | MedFC | 0.28 | 0.19 | n.s. | 0.22 | 0.73 | 0.66 | n.s | 0.87 | 0.66 | 0.62 | n.s | 0.83 |
| Frontal | MidFG l | 0.45 | 0.39 | n.s. | n.s. | 0.62 | 0.5 | n.s | n.s | 0.48 | 0.43 | n.s | n.s |
| Frontal | MidFG r | 0.47 | 0.41 | n.s. | n.s. | 0.63 | 0.46 | n.s | n.s | 0.49 | 0.38 | n.s | n.s |
| Frontal | PreCG l | 0.42 | 0.35 | n.s. | n.s. | 0.51 | 0.52 | n.s | n.s | 0.4 | n.s | n.s | n.s |
| Frontal | PreCG r | 0.41 | 0.34 | n.s. | n.s. | 0.56 | 0.59 | n.s | n.s | 0.47 | 0.55 | n.s | n.s |
| Frontal | SFG l | 0.43 | 0.35 | 0.26 | 0.36 | 0.61 | 0.38 | 0.45 | 0.54 | 0.51 | 0.34 | 0.42 | 0.48 |
| Frontal | SFG r | 0.44 | 0.36 | 0.29 | 0.37 | 0.63 | 0.54 | 0.54 | 0.67 | 0.53 | 0.46 | 0.49 | 0.6 |
| Frontal | SMA L | 0.35 | n.s | n.s. | n.s. | 0.52 | n.s | n.s | n.s | 0.43 | n.s | n.s | n.s |
| Frontal | SMA r | 0.34 | n.s | n.s. | n.s. | 0.6 | n.s | n.s | n.s | 0.55 | n.s | n.s | n.s |
| Temporal | aMTG l | 0.41 | 0.38 | 0.37 | 0.37 | 0.62 | 0.67 | 0.67 | 0.66 | 0.51 | 0.56 | 0.57 | 0.58 |
| Temporal | aMTG r | 0.48 | 0.45 | 0.44 | 0.44 | 0.66 | 0.64 | 0.68 | 0.64 | 0.5 | 0.51 | 0.54 | 0.52 |
| Temporal | aSTG l | 0.48 | n.s | 0.43 | 0.41 | 0.69 | n.s | 0.63 | 0.61 | 0.53 | n.s | 0.51 | 0.52 |
| Temporal | aSTG r | 0.49 | n.s | 0.44 | 0.41 | 0.73 | n.s | 0.68 | 0.73 | 0.57 | n.s | 0.56 | 0.62 |
| Temporal | HG l | 0.43 | 0.36 | 0.37 | n.s. | 0.65 | 0.67 | 0.7 | n.s | 0.53 | 0.62 | 0.61 | n.s |
| Temporal | HG r | 0.42 | 0.34 | 0.34 | n.s. | 0.68 | 0.7 | 0.68 | n.s | 0.58 | 0.64 | 0.6 | n.s |
| Temporal | pMTG l | 0.47 | 0.46 | 0.45 | 0.44 | 0.7 | 0.66 | 0.67 | 0.67 | 0.53 | 0.5 | 0.52 | 0.54 |
| Temporal | pMTG r | 0.5 | 0.48 | 0.48 | 0.45 | 0.7 | 0.69 | 0.68 | 0.72 | 0.53 | 0.53 | 0.51 | 0.57 |
| Temporal | pSTG l | 0.48 | 0.45 | 0.46 | 0.43 | 0.66 | 0.63 | 0.59 | 0.66 | 0.53 | 0.51 | 0.46 | 0.57 |
| Temporal | pSTG r | 0.51 | 0.48 | 0.47 | n.s. | 0.7 | 0.69 | 0.67 | 0.77 | 0.54 | 0.54 | 0.53 | 0.67 |
| Temporal | PT l | 0.47 | 0.45 | 0.45 | n.s. | 0.61 | 0.63 | 0.58 | n.s | 0.49 | 0.52 | 0.48 | n.s |
| Temporal | PT r | 0.48 | 0.43 | 0.42 | n.s. | 0.57 | 0.57 | 0.51 | n.s | 0.43 | 0.46 | 0.42 | n.s |
| Temporal | TP l | 0.46 | 0.41 | 0.4 | n.s. | 0.66 | 0.58 | 0.63 | n.s | 0.52 | 0.49 | 0.56 | n.s |
| Temporal | TP r | 0.49 | 0.43 | 0.44 | n.s. | 0.65 | 0.57 | 0.54 | n.s | 0.5 | 0.44 | 0.44 | n.s |
| Parietal | AG l | 0.32 | 0.22 | 0.21 | 0.27 | 0.56 | 0.61 | 0.62 | 0.46 | 0.49 | 0.56 | 0.59 | 0.41 |
| Parietal | AG r | 0.36 | 0.21 | n.s. | 0.24 | 0.68 | 0.68 | n.s | 0.61 | 0.59 | 0.63 | n.s | 0.58 |
| Parietal | Precuneous | 0.21 | n.s | n.s. | 0.15 | 0.65 | n.s | n.s | 0.72 | 0.6 | n.s | n.s | 0.65 |
| Parietal | pSMG l | 0.38 | 0.26 | 0.28 | 0.31 | 0.55 | 0.61 | 0.59 | 0.55 | 0.46 | 0.53 | 0.52 | 0.49 |
| Parietal | pSMG r | 0.39 | n.s | n.s. | 0.43 | 0.6 | n.s | n.s | n.s | 0.52 | n.s. | n.s | n.s |
| Limbic | AC | 0.46 | n.s | 0.28 | 0.4 | 0.72 | n.s | 0.67 | 0.71 | 0.57 | n.s | 0.61 | 0.6 |
| Limbic | PaCiG l | 0.46 | n.s | n.s. | 0.42 | 0.66 | n.s | n.s | 0.59 | 0.51 | 0.46 | n.s | 0.48 |
| Limbic | PaCiG r | 0.49 | n.s | n.s. | 0.45 | 0.64 | n.s | n.s | 0.56 | 0.46 | n.s | n.s | 0.45 |
| Insula | IC l | 0.47 | n.s | n.s | 0.36 | 0.69 | n.s | n.s | 0.58 | 0.54 | n.s | n.s | 0.51 |
| Insula | IC r | 0.46 | n.s | n.s | 0.32 | 0.63 | n.s | n.s | 0.66 | 0.5 | n.s | n.s | 0.6 |

**SI 8.-** Regression models. The left panel illustrates the specified regression model, while the right panel presents the corresponding bootstrapped estimates derived from the model.

| **1.      Right posterior supramarginal gyrus** |  |
| --- | --- |
| Call: |  |
| lm(formula = GlobalEfficiency_SC_RH.pSMG ~ AQ + VF + IF + VB + AQ:VF + AQ:VB, data = data1) | Parameter \| Coefficient \| 95% CI \| p |
|  | -------------------------------------------------- |
| Residuals: | (Intercept) \| 0.55 \| [ 0.15, 0.85] \| 0.009 |
| Min 1Q Median 3Q Max | AQ \| 2.24e-03 \| [-0.02, 0.03] \| 0.817 |
| -0.105032 -0.035729 0.000955 0.029990 0.117190 | VF \| -4.80e-03 \| [-0.01, 0.00] \| 0.142 |
|  | IF \| -0.01 \| [-0.02, 0.00] \| 0.008 |
| Coefficients: | VB \| 0.01 \| [-0.01, 0.05] \| 0.305 |
| Estimate Std. Error t value Pr(>\|t\|) | AQ:VF \| 3.59e-04 \| [ 0.00, 0.00] \| 0.069 |
| (Intercept) 0.5587266 0.1178895 4.739 3.32e-05 *** | AQ:VB \| -1.26e-03 \| [ 0.00, 0.00] \| 0.101 |
| AQ 0.0016784 0.0063835 0.263 0.79410 |  |
| VF -0.0045029 0.0041332 -1.089 0.28319 |  |
| IF -0.0114884 0.0041032 -2.800 0.00817 ** |  |
| VB 0.0122098 0.0107724 1.133 0.26453 |  |
| AQ:VF 0.0003409 0.0002201 1.549 0.13024 |  |
| AQ:VB -0.0011662 0.0005741 -2.031 0.04966 * |  |
| --- |  |
| Signif. codes: 0 ‘***’ 0.001 ‘**’ 0.01 ‘*’ 0.05 ‘.’ 0.1 ‘ ’ 1 |  |
|  |  |
| Residual standard error: 0.0593 on 36 degrees of freedom |  |
| (2 observations deleted due to missingness) |  |
| Multiple R-squared: 0.4388, Adjusted R-squared: 0.3453 |  |
| F-statistic: 4.692 on 6 and 36 DF, p-value: 0.001275 |  |
|  |  |
| **2.      Precuneus** |  |
| Call: |  |
| lm(formula = GlobalEfficiency_SC_Precuneus ~ AQ + MaTr + VP + |  |
| AQ:VP, data = data1) | Parameter \| Coefficient \| 95% CI \| p |
|  | --------------------------------------------------- |
| Residuals: | (Intercept) \| 1.22 \| [ 0.56, 2.26] \| < .001 |
| Min 1Q Median 3Q Max | AQ \| -0.05 \| [-0.10, -0.02] \| 0.005 |
| -0.27140 -0.12662 -0.01555 0.10428 0.31818 | MaTr \| -0.05 \| [-0.08, -0.02] \| < .001 |
|  | VP \| -0.03 \| [-0.13, 0.02] \| 0.308 |
| Coefficients: | AQ:VP \| 3.74e-03 \| [ 0.00, 0.01] \| 0.009 |
| Estimate Std. Error t value Pr(>\|t\|) |  |
| (Intercept) 1.130117 0.397442 2.843 0.006996 ** |  |
| AQ -0.044520 0.019975 -2.229 0.031510 * |  |
| MaTr -0.048671 0.012734 -3.822 0.000453 *** |  |
| VP -0.026912 0.033196 -0.811 0.422342 |  |
| AQ:VP 0.003582 0.001748 2.049 0.047093 * |  |
| --- |  |
| Signif. codes: 0 ‘***’ 0.001 ‘**’ 0.01 ‘*’ 0.05 ‘.’ 0.1 ‘ ’ 1 |  |
|  |  |
| Residual standard error: 0.164 on 40 degrees of freedom |  |
| Multiple R-squared: 0.3697, Adjusted R-squared: 0.3066 |  |
| F-statistic: 5.865 on 4 and 40 DF, p-value: 0.0008227 |  |
|  |  |
| **3.      Left anterior superior temporal gyrus** |  |
| Call: |  |
| lm(formula = LocalEfficiency_SC_LH.aSTG ~ AQ + VF + SE + VB + AQ:VF + AQ:SE, data = data1) |  |
|  | Parameter \| Coefficient \| 95% CI \| p |
| Residuals: | -------------------------------------------------- |
| Min 1Q Median 3Q Max | (Intercept) \| -0.27 \| [-1.00, 1.06] \| 0.627 |
| -0.55008 -0.05007 0.02699 0.10105 0.41456 | AQ \| 0.03 \| [-0.05, 0.07] \| 0.354 |
|  | VF \| 0.04 \| [ 0.01, 0.07] \| 0.020 |
| Coefficients: | SE \| -0.05 \| [-0.16, 0.00] \| 0.037 |
| Estimate Std. Error t value Pr(>\|t\|) | VB \| 0.04 \| [ 0.00, 0.07] \| 0.040 |
| (Intercept) -0.379965 0.312300 -1.217 0.23143 | AQ:VF \| -1.83e-03 \| [ 0.00, 0.00] \| 0.039 |
| AQ 0.034033 0.017292 1.968 0.05658 . | AQ:SE \| 2.31e-03 \| [ 0.00, 0.01] \| 0.104 |
| VF 0.043686 0.012899 3.387 0.00169 ** |  |
| SE -0.042323 0.023614 -1.792 0.08128 . |  |
| VB 0.033693 0.014776 2.280 0.02845 * |  |
| AQ:VF -0.001925 0.000688 -2.798 0.00812 ** |  |
| AQ:SE 0.001958 0.001324 1.479 0.14756 |  |
| --- |  |
| Signif. codes: 0 ‘***’ 0.001 ‘**’ 0.01 ‘*’ 0.05 ‘.’ 0.1 ‘ ’ 1 |  |
|  |  |
| Residual standard error: 0.1959 on 37 degrees of freedom |  |
| (1 observation deleted due to missingness) |  |
| Multiple R-squared: 0.4398, Adjusted R-squared: 0.349 |  |
| F-statistic: 4.842 on 6 and 37 DF, p-value: 0.0009715 |  |
|  |  |
| **4.      Left posterior middle temporal gyrus** |  |
| Call: |  |
| lm(formula = LocalEfficiency_SC_LH.pMTG ~ AQ + VF + VB + AQ:VF + AQ:VB, data = data1) |  |
|  |  |
| Residuals: |  |
| Min 1Q Median 3Q Max | Parameter \| Coefficient \| 95% CI \| p |
| -0.245342 -0.057495 -0.000008 0.062955 0.288504 | --------------------------------------------------- |
|  | (Intercept) \| 1.46 \| [ 1.19, 1.90] \| < .001 |
| Coefficients: | AQ \| -0.04 \| [-0.07, -0.02] \| 0.006 |
| Estimate Std. Error t value Pr(>\|t\|) | VF \| -0.02 \| [-0.04, -0.01] \| 0.012 |
| (Intercept) 1.4676257 0.2218796 6.615 9.3e-08 *** | VB \| -0.01 \| [-0.06, 0.02] \| 0.448 |
| AQ -0.0432722 0.0126376 -3.424 0.00152 ** | AQ:VF \| 9.22e-04 \| [ 0.00, 0.00] \| 0.062 |
| VF -0.0241335 0.0083532 -2.889 0.00642 ** | AQ:VB \| 1.90e-03 \| [ 0.00, 0.00] \| 0.193 |
| VB -0.0157471 0.0217356 -0.724 0.47333 |  |
| AQ:VF 0.0009023 0.0004406 2.048 0.04770 * |  |
| AQ:VB 0.0018873 0.0011631 1.623 0.11315 |  |
| --- |  |
| Signif. codes: 0 ‘***’ 0.001 ‘**’ 0.01 ‘*’ 0.05 ‘.’ 0.1 ‘ ’ 1 |  |
|  |  |
| Residual standard error: 0.1206 on 37 degrees of freedom |  |
| (2 observations deleted due to missingness) |  |
| Multiple R-squared: 0.4167, Adjusted R-squared: 0.3379 |  |
| F-statistic: 5.286 on 5 and 37 DF, p-value: 0.0009211 |  |
|  |  |
| **5.      Right frontal pole** |  |
| Call: |  |
| lm(formula = LocalEfficiency_SC_RH.FP ~ AQ + Tower.of.London + nback + AQ:Tower.of.London + AQ:nback, data = data1) |  |
|  |  |
| Residuals: | Parameter \| Coefficient \| 95% CI \| p |
| Min 1Q Median 3Q Max | -------------------------------------------------------- |
| -0.39021 -0.11717 0.04457 0.12061 0.45271 | (Intercept) \| -1.46 \| [-4.66, 0.28] \| 0.092 |
|  | AQ \| 0.17 \| [ 0.03, 0.37] \| 0.023 |
| Coefficients: | Tower.of.London \| 0.11 \| [-0.03, 0.18] \| 0.131 |
| Estimate Std. Error t value Pr(>\|t\|) | nback \| 8.10e-03 \| [ 0.00, 0.03] \| 0.105 |
| (Intercept) -1.6305550 1.0377557 -1.571 0.124642 | AQ:Tower.of.London \| -7.30e-03 \| [-0.01, 0.00] \| 0.071 |
| AQ 0.1627610 0.0733516 2.219 0.032707 * | AQ:nback \| -6.65e-04 \| [ 0.00, 0.00] \| 0.085 |
| Tower.of.London 0.1108285 0.0361819 3.063 0.004070 ** |  |
| nback 0.0083107 0.0066642 1.247 0.220204 |  |
| AQ:Tower.of.London -0.0076245 0.0020420 -3.734 0.000633 *** |  |
| AQ:nback -0.0006090 0.0004462 -1.365 0.180545 |  |
| --- |  |
| Signif. codes: 0 ‘***’ 0.001 ‘**’ 0.01 ‘*’ 0.05 ‘.’ 0.1 ‘ ’ 1 |  |
|  |  |
| Residual standard error: 0.1912 on 37 degrees of freedom |  |
| (2 observations deleted due to missingness) |  |
| Multiple R-squared: 0.4287, Adjusted R-squared: 0.3515 |  |
| F-statistic: 5.553 on 5 and 37 DF, p-value: 0.0006508 |  |
|  |  |
| **6.      Right anterior middle temporal gyrus** |  |
| Call: |  |
| lm(formula = LocalEfficiency_SC_RH.aMTG ~ AQ + gonogo + nback + Digit.span + AQ:gonogo, data = data1) |  |
|  |  |
| Residuals: |  |
| Min 1Q Median 3Q Max |  |
| -0.30638 -0.05938 0.01373 0.08915 0.29352 |  |
|  | Parameter \| Coefficient \| 95% CI \| p |
| Coefficients: | -------------------------------------------------- |
| Estimate Std. Error t value Pr(>\|t\|) | (Intercept) \| 4.54 \| [-0.30, 8.94] \| 0.067 |
| (Intercept) 4.9355561 2.0824550 2.370 0.0238 * | AQ \| -0.27 \| [-0.51, 0.00] \| 0.048 |
| AQ -0.2804917 0.1117176 -2.511 0.0171 * | gonogo \| -0.01 \| [-0.03, 0.00] \| 0.089 |
| gonogo -0.0154862 0.0068651 -2.256 0.0308 * | nback \| 4.10e-03 \| [ 0.00, 0.01] \| 0.071 |
| nback 0.0044089 0.0019267 2.288 0.0287 * | Digit.span \| -0.01 \| [-0.03, 0.01] \| 0.298 |
| Digit.span -0.0111198 0.0084004 -1.324 0.1947 | AQ:gonogo \| 8.37e-04 \| [ 0.00, 0.00] \| 0.049 |
| AQ:gonogo 0.0008812 0.0003619 2.435 0.0205 * |  |
| --- |  |
| Signif. codes: 0 ‘***’ 0.001 ‘**’ 0.01 ‘*’ 0.05 ‘.’ 0.1 ‘ ’ 1 |  |
|  |  |
| Residual standard error: 0.1352 on 33 degrees of freedom |  |
| (6 observations deleted due to missingness) |  |
| Multiple R-squared: 0.3557, Adjusted R-squared: 0.2581 |  |
| F-statistic: 3.644 on 5 and 33 DF, p-value: 0.009814 |  |

**SI 9.-** Global Efficiency for Social Communication Network.

| **Global Efficiency** | | | | | | |
| --- | --- | --- | --- | --- | --- | --- |
| **ROI** | **All** | | **Autistic** | | **Non-Autistic** | |
|  | **beta** | **dof** | **beta** | **dof** | **beta** | **dof** |
| network | 0.43 | 70 | 0.44 | 31 | 0.41 | 36 |
| AC | 0.35 | 70 | 0.39 | 31 |  |  |
| AG l | 0.45 | 70 | 0.44 | 31 | 0.45 | 36 |
| AG r | 0.4 | 70 | 0.39 | 31 | 0.41 | 36 |
| aMTG l | 0.5 | 70 | 0.47 | 31 | 0.55 | 36 |
| aMTG r | 0.43 | 70 | 0.49 | 31 | 0.4 | 36 |
| aSTG l | 0.42 | 70 |  |  |  |  |
| aSTG r | 0.44 | 70 |  |  |  |  |
| FP l | 0.4 | 70 | 0.38 | 31 | 0.41 | 36 |
| FP r | 0.41 | 70 | 0.4 | 31 | 0.43 | 36 |
| HG l | 0.43 | 70 | 0.44 | 31 | 0.41 | 36 |
| HG r | 0.42 | 70 | 0.42 | 31 | 0.4 | 36 |
| IC l | 0.5 | 70 | 0.46 | 31 | 0.54 | 36 |
| IC r | 0.57 | 70 | 0.56 | 31 | 0.58 | 36 |
| IFG oper l | 0.41 | 70 | 0.47 | 31 | 0.36 | 36 |
| IFG oper r | 0.53 | 70 | 0.53 | 31 | 0.55 | 36 |
| IFGor l | 0.57 | 70 | 0.64 | 31 | 0.48 | 36 |
| IFGor r |  |  |  |  |  |  |
| IFG tri l | 0.45 | 70 | 0.48 | 31 | 0.41 | 36 |
| IFG tri r | 0.48 | 70 | 0.53 | 31 | 0.44 | 36 |
| MedFC |  |  |  |  |  |  |
| MidFG l | 0.4 | 70 | 0.42 | 31 | 0.38 | 36 |
| MidFG r | 0.38 | 70 | 0.43 | 31 | 0.33 | 36 |
| PaCiG l | 0.41 | 70 | 0.39 | 31 | 0.45 | 36 |
| PaCiG r | 0.34 | 70 |  |  |  |  |
| pMTG l | 0.39 | 70 | 0.35 | 31 | 0.43 | 36 |
| pMTG r | 0.42 | 70 | 0.37 | 31 | 0.46 | 36 |
| PreCG l | 0.41 | 70 | 0.45 | 31 |  |  |
| PreCG r | 0.39 | 70 | 0.38 | 31 | 0.4 | 36 |
| Precuneus |  |  |  |  |  |  |
| pSMG l | 0.46 | 70 | 0.44 | 31 | 0.49 | 36 |
| pSMG r | 0.46 | 70 | 0.48 | 31 | 0.43 | 36 |
| pSTG l | 0.42 | 70 | 0.4 | 31 | 0.43 | 36 |
| pSTG r | 0.44 | 70 |  |  | 0.42 | 36 |
| PT l | 0.44 | 70 | 0.42 | 31 | 0.43 | 36 |
| PT r | 0.5 | 70 | 0.53 | 31 | 0.45 | 36 |
| SFG l | 0.45 | 70 | 0.49 | 31 | 0.41 | 36 |
| SFG r | 0.44 | 70 | 0.47 | 31 | 0.42 | 36 |
| SMA L | 0.43 | 70 | 0.51 | 31 |  |  |
| SMA r | 0.42 | 70 | 0.45 | 31 | 0.4 | 36 |
| TP l | 0.48 | 70 |  |  | 0.51 | 36 |
| TP r | 0.42 | 70 |  |  | 0.42 | 36 |

**SI 10.-** Local Efficiency for Social Communication Network.

| **Local Efficiency** | | | | | | |
| --- | --- | --- | --- | --- | --- | --- |
| **ROI** | **All** | | **Autistic** | | **Non-Autistic** | |
|  | **beta** | **dof** | **beta** | **dof** | **beta** | **dof** |
| network | 0.64 | 70 | 0.58 | 31 | 0.59 | 36 |
| AC |  |  |  |  |  |  |
| AG l | 0.62 | 68 |  |  |  |  |
| AG r | 0.75 | 68 |  |  | 0.74 | 35 |
| aMTG l | 0.49 | 68 |  |  |  |  |
| aMTG r | 0.8 | 65 |  |  |  |  |
| aSTG l | 0.86 | 52 |  |  |  |  |
| aSTG r |  |  |  |  |  |  |
| FP l | 0.66 | 69 |  |  |  |  |
| FP r | 0.68 | 70 |  |  |  |  |
| HG l | 0.89 | 66 | 0.88 | 29 |  |  |
| HG r | 0.92 | 68 | 0.89 | 30 |  |  |
| IC l | 0.68 | 62 |  |  |  |  |
| IC r |  |  |  |  |  |  |
| IFG oper l | 0.73 | 70 |  |  | 0.78 | 36 |
| IFG oper r |  |  |  |  |  |  |
| IFGor l |  |  |  |  |  |  |
| IFGor r |  |  |  |  |  |  |
| IFG tri l | 0.81 | 68 | 0.84 | 30 |  |  |
| IFG tri r | 0.82 | 62 |  |  |  |  |
| MedFC |  |  |  |  |  |  |
| MidFG l | 0.64 | 70 | 0.67 | 31 |  |  |
| MidFG r | 0.71 | 70 |  |  | 0.79 | 36 |
| PaCiG l | 1.03 | 63 |  |  |  |  |
| PaCiG r |  |  |  |  |  |  |
| pMTG l | 0.66 | 68 |  |  |  |  |
| pMTG r | 0.73 | 69 |  |  | 0.84 | 36 |
| PreCG l | 0.63 | 68 |  |  | 0.97 | 34 |
| PreCG r | 0.81 | 69 |  |  |  |  |
| Precuneus |  |  |  |  |  |  |
| pSMG l | 0.69 | 70 |  |  | 0.67 | 36 |
| pSMG r | 0.57 | 69 |  |  |  |  |
| pSTG l | 0.56 | 67 |  |  |  |  |
| pSTG r |  |  |  |  |  |  |
| PT l | 0.81 | 68 | 0.73 | 30 |  |  |
| PT r | 0.78 | 70 | 0.61 | 31 |  |  |
| SFG l | 0.64 | 70 | 0.66 | 31 |  |  |
| SFG r |  |  |  |  |  |  |
| SMA L | 0.72 | 65 |  |  | 1.01 | 35 |
| SMA r | 0.75 | 68 |  |  |  |  |
| TP l |  |  |  |  |  |  |
| TP r |  |  |  |  |  |  |

**SI 11.-** Clustering Efficiency for Social Communication Network.

| **Clustering Coefficient** | | | | | | |
| --- | --- | --- | --- | --- | --- | --- |
| **ROI** | **All** | | **Autistic** | | **Non-Autistic** | |
|  | **beta** | **dof** | **beta** | **dof** | **beta** | **dof** |
| network | 0.52 | 70 |  |  | 0.53 | 44 |
| AC |  |  |  |  |  |  |
| AG l | 0.52 | 68 |  |  |  |  |
| AG r | 0.69 | 68 |  |  |  |  |
| aMTG l |  |  |  |  |  |  |
| aMTG r | 0.68 | 65 |  |  |  |  |
| aSTG l | 0.69 | 52 |  |  |  |  |
| aSTG r |  |  |  |  |  |  |
| FP l | 0.53 | 69 |  |  |  |  |
| FP r | 0.55 | 70 |  |  |  |  |
| HG l | 0.76 | 66 |  |  |  |  |
| HG r | 0.85 | 68 |  |  |  |  |
| IC l |  |  |  |  |  |  |
| IC r |  |  |  |  |  |  |
| IFG oper l | 0.57 | 70 |  |  |  |  |
| IFG oper r |  |  |  |  |  |  |
| IFGor l |  |  |  |  |  |  |
| IFGor r |  |  |  |  |  |  |
| IFG tri l | 0.56 | 68 |  |  |  |  |
| IFG tri r |  |  |  |  |  |  |
| MedFC |  |  |  |  |  |  |
| MidFG l | 0.44 | 70 |  |  |  |  |
| MidFG r | 0.64 | 70 |  |  |  |  |
| PaCiG l | 0.98 | 63 |  |  |  |  |
| PaCiG r |  |  |  |  |  |  |
| pMTG l | 0.57 | 68 |  |  | 0.78 | 43 |
| pMTG r | 0.61 | 69 |  |  |  |  |
| PreCG l | 0.52 | 68 |  |  |  |  |
| PreCG r | 0.7 | 69 |  |  |  |  |
| Precuneus |  |  |  |  |  |  |
| pSMG l | 0.58 | 70 |  |  |  |  |
| pSMG r | 0.5 | 69 |  |  |  |  |
| pSTG l |  |  |  |  |  |  |
| pSTG r |  |  |  |  |  |  |
| PT l | 0.77 | 68 |  |  |  |  |
| PT r | 0.54 | 70 |  |  |  |  |
| SFG l |  |  |  |  |  |  |
| SFG r |  |  |  |  |  |  |
| SMA L | 0.69 | 65 |  |  |  |  |
| SMA r | 0.59 | 68 |  |  |  |  |
| TP l |  |  |  |  |  |  |
| TP r |  |  |  |  |  |  |

**SI 12.-** **Regression models for the Autism Quotient replication analysis.** The left panel illustrates the specified regression model, and the right panel displays the corresponding bootstrapped parameter estimates derived from the model.

| **Global Efficiency Left posterior Supramarginal Gyrus** | |
| --- | --- |
| Linear mixed model fit by REML. t-tests use Satterthwaite's method ['lmerModLmerTest'] Formula: GlobalEfficiency_SC_LH.pSMG ~ AQ + sex + (1 \| site_id) + (1 \| dx_group)  Data: data_ABIDE_AQ  REML criterion at convergence: -167.9  Scaled residuals:   Min 1Q Median 3Q Max  -2.14557 -0.56476 0.08706 0.64902 1.97558   Random effects:  Groups Name Variance Std.Dev.  site_id (Intercept) 9.011e-05 0.009493  dx_group (Intercept) 0.000e+00 0.000000  Residual 4.262e-03 0.065282 Number of obs: 73, groups: site_id, 3; dx_group, 2  Fixed effects:  Estimate Std. Error df t value Pr(>\|t\|)  (Intercept) 0.5049946 0.0336293 49.2896256 15.017 <2e-16 *** AQ -0.0019444 0.0007516 68.8670194 -2.587 0.0118 *  sexmale 0.0080435 0.0309187 63.4489676 0.260 0.7956  --- Signif. codes: 0 ‘***’ 0.001 ‘**’ 0.01 ‘*’ 0.05 ‘.’ 0.1 ‘ ’ 1  Correlation of Fixed Effects:  (Intr) AQ  AQ -0.429  sexmale -0.834 -0.054 optimizer (nloptwrap) convergence code: 0 (OK) boundary (singular) fit: see help('isSingular') | # Fixed Effects  Parameter \| Coefficient \| 95% CI \| p --------------------------------------------------- (Intercept) \| 0.50 \| [ 0.44, 0.57] \| < .001 AQ \| -1.94e-03 \| [ 0.00, 0.00] \| 0.010  sexmale \| 8.13e-03 \| [-0.05, 0.07] \| 0.797 |
| **Global Efficiency Right Inferior Frontal Gyrus opercularis** | |
| Linear mixed model fit by REML. t-tests use Satterthwaite's method ['lmerModLmerTest'] Formula: GlobalEfficiency_SC_RH.IFGop ~ AQ + sex + (1 \| site_id) + (1 \| dx_group)  Data: data_ABIDE_AQ  REML criterion at convergence: -116.2  Scaled residuals:   Min 1Q Median 3Q Max  -4.0897 -0.3536 0.1729 0.5841 1.6591   Random effects:  Groups Name Variance Std.Dev.  site_id (Intercept) 0.0002008 0.01417   dx_group (Intercept) 0.0000000 0.00000   Residual 0.0089168 0.09443  Number of obs: 73, groups: site_id, 3; dx_group, 2  Fixed effects:  Estimate Std. Error df t value Pr(>\|t\|)  (Intercept) 0.408121 0.048715 47.411514 8.378 6.54e-11 *** AQ -0.002354 0.001087 68.704485 -2.165 0.0339 *  sexmale 0.061585 0.044762 63.127946 1.376 0.1737  --- Signif. codes: 0 ‘***’ 0.001 ‘**’ 0.01 ‘*’ 0.05 ‘.’ 0.1 ‘ ’ 1  Correlation of Fixed Effects:  (Intr) AQ  AQ -0.428  sexmale -0.833 -0.055 optimizer (nloptwrap) convergence code: 0 (OK) boundary (singular) fit: see help('isSingular') | # Fixed Effects  Parameter \| Coefficient \| 95% CI \| p --------------------------------------------------- (Intercept) \| 0.41 \| [ 0.31, 0.50] \| < .001 AQ \| -2.34e-03 \| [ 0.00, 0.00] \| 0.034  sexmale \| 0.06 \| [-0.03, 0.15] \| 0.170 |
| **Local Efficiency Left Heschel Gyrus** | |
| Linear mixed model fit by REML. t-tests use Satterthwaite's method ['lmerModLmerTest'] Formula: LocalEfficiency_SC_LH.HG ~ AQ + sex + (1 \| site_id) + (1 \| dx_group)  Data: data_ABIDE_AQ  REML criterion at convergence: 8.1  Scaled residuals:   Min 1Q Median 3Q Max  -3.3917 -0.2611 0.1351 0.5792 1.4547   Random effects:  Groups Name Variance Std.Dev.  site_id (Intercept) 0.005112 0.0715   dx_group (Intercept) 0.000000 0.0000   Residual 0.051253 0.2264  Number of obs: 69, groups: site_id, 3; dx_group, 2  Fixed effects:  Estimate Std. Error df t value Pr(>\|t\|)  (Intercept) 0.582715 0.124925 36.085135 4.665 4.15e-05 *** AQ 0.006943 0.002722 64.349015 2.551 0.0131 *  sexmale 0.054985 0.109671 65.922168 0.501 0.6178  --- Signif. codes: 0 ‘***’ 0.001 ‘**’ 0.01 ‘*’ 0.05 ‘.’ 0.1 ‘ ’ 1  Correlation of Fixed Effects:  (Intr) AQ  AQ -0.408  sexmale -0.793 -0.066 optimizer (nloptwrap) convergence code: 0 (OK) boundary (singular) fit: see help('isSingular') | # Fixed Effects  Parameter \| Coefficient \| 95% CI \| p -------------------------------------------------- (Intercept) \| 0.58 \| [ 0.33, 0.83] \| < .001 AQ \| 6.93e-03 \| [ 0.00, 0.01] \| 0.010  sexmale \| 0.06 \| [-0.16, 0.27] \| 0.611 |
| **Local Efficiency Left Planum Temporale** | |
| Linear mixed model fit by REML. t-tests use Satterthwaite's method ['lmerModLmerTest'] Formula: LocalEfficiency_SC_LH.PT ~ AQ + sex + (1 \| site_id) + (1 \| dx_group)  Data: data_ABIDE_AQ  REML criterion at convergence: -13.3  Scaled residuals:   Min 1Q Median 3Q Max  -3.6961 -0.3404 0.1486 0.6653 1.4894   Random effects:  Groups Name Variance Std.Dev.  site_id (Intercept) 0.0000 0.000   dx_group (Intercept) 0.0000 0.000   Residual 0.0388 0.197  Number of obs: 71, groups: site_id, 3; dx_group, 2  Fixed effects:  Estimate Std. Error df t value Pr(>\|t\|)  (Intercept) 0.694759 0.099363 68.000000 6.992 1.47e-09 *** AQ 0.005341 0.002346 68.000000 2.277 0.0259 *  sexmale -0.025504 0.091450 68.000000 -0.279 0.7812  --- Signif. codes: 0 ‘***’ 0.001 ‘**’ 0.01 ‘*’ 0.05 ‘.’ 0.1 ‘ ’ 1  Correlation of Fixed Effects:  (Intr) AQ  AQ -0.463  sexmale -0.834 -0.044 optimizer (nloptwrap) convergence code: 0 (OK) boundary (singular) fit: see help('isSingular') | # Fixed Effects  Parameter \| Coefficient \| 95% CI \| p -------------------------------------------------- (Intercept) \| 0.69 \| [ 0.50, 0.89] \| < .001 AQ \| 5.35e-03 \| [ 0.00, 0.01] \| 0.022  sexmale \| -0.03 \| [-0.20, 0.16] \| 0.776 |
| **Local Efficiency Left anterior Superior Temporal Gyrus** | |
| Linear mixed model fit by REML. t-tests use Satterthwaite's method ['lmerModLmerTest'] Formula: ClusteringCoefficient_SC_LH.aSTG ~ AQ + sex + (1 \| site_id) + (1 \| dx_group)  Data: data_ABIDE_AQ  REML criterion at convergence: 17.1  Scaled residuals:   Min 1Q Median 3Q Max  -2.15926 -0.43827 0.00217 0.57885 1.77534   Random effects:  Groups Name Variance Std.Dev.  site_id (Intercept) 0.00000 0.0000   dx_group (Intercept) 0.00000 0.0000   Residual 0.06212 0.2492  Number of obs: 55, groups: site_id, 3; dx_group, 2  Fixed effects:  Estimate Std. Error df t value Pr(>\|t\|)  (Intercept) 0.391497 0.135112 52.000000 2.898 0.00549 ** AQ 0.006451 0.003164 52.000000 2.039 0.04655 *  sexmale 0.056351 0.130696 52.000000 0.431 0.66814  --- Signif. codes: 0 ‘***’ 0.001 ‘**’ 0.01 ‘*’ 0.05 ‘.’ 0.1 ‘ ’ 1  Correlation of Fixed Effects:  (Intr) AQ  AQ -0.386  sexmale -0.825 -0.140 optimizer (nloptwrap) convergence code: 0 (OK) boundary (singular) fit: see help('isSingular') | # Fixed Effects  Parameter \| Coefficient \| 95% CI \| p ------------------------------------------------- (Intercept) \| 0.39 \| [ 0.12, 0.66] \| 0.006 AQ \| 6.55e-03 \| [ 0.00, 0.01] \| 0.041 sexmale \| 0.05 \| [-0.20, 0.32] \| 0.681 |
| **Local Efficiency Right Angular Gyrus** | |
| Linear mixed model fit by REML. t-tests use Satterthwaite's method ['lmerModLmerTest'] Formula: LocalEfficiency_SC_RH.AG ~ AQ + sex + (1 \| site_id) + (1 \| dx_group)  Data: data_ABIDE_AQ  REML criterion at convergence: -30.4  Scaled residuals:   Min 1Q Median 3Q Max  -3.5388 -0.5491 0.2153 0.6330 1.9859   Random effects:  Groups Name Variance Std.Dev.  site_id (Intercept) 0.00000 0.0000   dx_group (Intercept) 0.01487 0.1219   Residual 0.02922 0.1709  Number of obs: 71, groups: site_id, 3; dx_group, 2  Fixed effects:  Estimate Std. Error df t value Pr(>\|t\|)  (Intercept) 0.545523 0.134414 4.160244 4.059 0.0142 * AQ 0.005986 0.002845 58.090166 2.104 0.0397 * sexmale -0.008162 0.079958 67.239691 -0.102 0.9190  --- Signif. codes: 0 ‘***’ 0.001 ‘**’ 0.01 ‘*’ 0.05 ‘.’ 0.1 ‘ ’ 1  Correlation of Fixed Effects:  (Intr) AQ  AQ -0.507  sexmale -0.585 0.061 optimizer (nloptwrap) convergence code: 0 (OK) boundary (singular) fit: see help('isSingular') | # Fixed Effects  Parameter \| Coefficient \| 95% CI \| p -------------------------------------------------- (Intercept) \| 0.55 \| [ 0.28, 0.81] \| < .001 AQ \| 5.98e-03 \| [ 0.00, 0.01] \| 0.048  sexmale \| -8.88e-03 \| [-0.17, 0.15] \| 0.914 |
| **Local Efficiency Right Middle Frontal Gyrus** | |
| Linear mixed model fit by REML. t-tests use Satterthwaite's method ['lmerModLmerTest'] Formula: LocalEfficiency_SC_RH.MFG ~ AQ + sex + (1 \| site_id) + (1 \| dx_group)  Data: data_ABIDE_AQ  REML criterion at convergence: -9.1  Scaled residuals:   Min 1Q Median 3Q Max  -3.1961 -0.6108 0.2251 0.6249 1.6892   Random effects:  Groups Name Variance Std.Dev.  site_id (Intercept) 0.001546 0.03932   dx_group (Intercept) 0.010182 0.10091   Residual 0.039958 0.19989  Number of obs: 73, groups: site_id, 3; dx_group, 2  Fixed effects:  Estimate Std. Error df t value Pr(>\|t\|)  (Intercept) 0.445680 0.141732 7.090990 3.145 0.0160 * AQ 0.007819 0.003252 36.947863 2.405 0.0213 * sexmale 0.050207 0.096069 66.712447 0.523 0.6030  --- Signif. codes: 0 ‘***’ 0.001 ‘**’ 0.01 ‘*’ 0.05 ‘.’ 0.1 ‘ ’ 1  Correlation of Fixed Effects:  (Intr) AQ  AQ -0.535  sexmale -0.656 0.035 | # Fixed Effects  Parameter \| Coefficient \| 95% CI \| p ------------------------------------------------- (Intercept) \| 0.23 \| [-0.07, 0.52] \| 0.123 AQ \| 9.80e-03 \| [ 0.00, 0.02] \| 0.005 sexmale \| 0.08 \| [-0.10, 0.26] \| 0.397 |
| **Clustering Coefficient Left Heschel Gyrus** | |
| Linear mixed model fit by REML. t-tests use Satterthwaite's method ['lmerModLmerTest'] Formula: ClusteringCoefficient_SC_LH.HG ~ AQ + sex + (1 \| site_id) + (1 \| dx_group)  Data: data_ABIDE_AQ  REML criterion at convergence: 12.3  Scaled residuals:   Min 1Q Median 3Q Max  -2.9831 -0.4068 -0.1227 0.7170 1.7265   Random effects:  Groups Name Variance Std.Dev.  site_id (Intercept) 0.004022 0.06342   dx_group (Intercept) 0.000000 0.00000   Residual 0.054937 0.23439  Number of obs: 69, groups: site_id, 3; dx_group, 2  Fixed effects:  Estimate Std. Error df t value Pr(>\|t\|)  (Intercept) 0.472097 0.127094 40.645205 3.715 0.000612 *** AQ 0.007370 0.002817 64.485660 2.616 0.011060 *  sexmale 0.067225 0.113092 65.544314 0.594 0.554274  --- Signif. codes: 0 ‘***’ 0.001 ‘**’ 0.01 ‘*’ 0.05 ‘.’ 0.1 ‘ ’ 1  Correlation of Fixed Effects:  (Intr) AQ  AQ -0.417  sexmale -0.804 -0.064 optimizer (nloptwrap) convergence code: 0 (OK) boundary (singular) fit: see help('isSingular') | # Fixed Effects  Parameter \| Coefficient \| 95% CI \| p -------------------------------------------------- (Intercept) \| 0.47 \| [ 0.22, 0.72] \| < .001 AQ \| 7.37e-03 \| [ 0.00, 0.01] \| 0.008  sexmale \| 0.07 \| [-0.15, 0.29] \| 0.552 |
| **Clustering Coefficient Left Middle Frontal Gyrus** | |
| Linear mixed model fit by REML. t-tests use Satterthwaite's method ['lmerModLmerTest'] Formula: ClusteringCoefficient_SC_LH.MFG ~ AQ + sex + (1 \| site_id) + (1 \| dx_group)  Data: data_ABIDE_AQ  REML criterion at convergence: -53.5  Scaled residuals:   Min 1Q Median 3Q Max  -3.1648 -0.4737 0.0122 0.5828 2.5851   Random effects:  Groups Name Variance Std.Dev.  site_id (Intercept) 0.00000 0.0000   dx_group (Intercept) 0.00000 0.0000   Residual 0.02209 0.1486  Number of obs: 73, groups: site_id, 3; dx_group, 2  Fixed effects:  Estimate Std. Error df t value Pr(>\|t\|)  (Intercept) 0.447361 0.074433 70.000000 6.010 7.51e-08 *** AQ 0.003552 0.001709 70.000000 2.079 0.0413 *  sexmale -0.037342 0.068943 70.000000 -0.542 0.5898  --- Signif. codes: 0 ‘***’ 0.001 ‘**’ 0.01 ‘*’ 0.05 ‘.’ 0.1 ‘ ’ 1  Correlation of Fixed Effects:  (Intr) AQ  AQ -0.450  sexmale -0.841 -0.045 optimizer (nloptwrap) convergence code: 0 (OK) boundary (singular) fit: see help('isSingular') | # Fixed Effects  Parameter \| Coefficient \| 95% CI \| p -------------------------------------------------- (Intercept) \| 0.45 \| [ 0.30, 0.59] \| < .001 AQ \| 3.57e-03 \| [ 0.00, 0.01] \| 0.039  sexmale \| -0.04 \| [-0.17, 0.10] \| 0.598 |
| **Clustering Coefficient Right Angular Gyrus** | |
| Linear mixed model fit by REML. t-tests use Satterthwaite's method ['lmerModLmerTest'] Formula: ClusteringCoefficient_SC_RH.AG ~ AQ + sex + (1 \| site_id) + (1 \| dx_group)  Data: data_ABIDE_AQ  REML criterion at convergence: -32.4  Scaled residuals:   Min 1Q Median 3Q Max  -2.5962 -0.6086 -0.1310 0.5709 3.0671   Random effects:  Groups Name Variance Std.Dev.  site_id (Intercept) 0.00000 0.0000   dx_group (Intercept) 0.01308 0.1144   Residual 0.02841 0.1686  Number of obs: 71, groups: site_id, 3; dx_group, 2  Fixed effects:  Estimate Std. Error df t value Pr(>\|t\|)  (Intercept) 0.370367 0.129764 4.470234 2.854 0.0406 * AQ 0.005956 0.002796 55.867070 2.130 0.0376 * sexmale -0.004773 0.078832 67.260297 -0.061 0.9519  --- Signif. codes: 0 ‘***’ 0.001 ‘**’ 0.01 ‘*’ 0.05 ‘.’ 0.1 ‘ ’ 1  Correlation of Fixed Effects:  (Intr) AQ  AQ -0.516  sexmale -0.597 0.060 optimizer (nloptwrap) convergence code: 0 (OK) boundary (singular) fit: see help('isSingular') | # Fixed Effects  Parameter \| Coefficient \| 95% CI \| p ------------------------------------------------- (Intercept) \| 0.37 \| [ 0.11, 0.63] \| 0.006 AQ \| 5.93e-03 \| [ 0.00, 0.01] \| 0.041 sexmale \| -5.57e-03 \| [-0.16, 0.15] \| 0.943 |
| **Clustering Coefficient Right Middle Frontal Gyrus** | |
| Linear mixed model fit by REML. t-tests use Satterthwaite's method ['lmerModLmerTest'] Formula: ClusteringCoefficient_SC_RH.MFG ~ AQ + sex + (1 \| site_id) + (1 \| dx_group)  Data: data_ABIDE_AQ  REML criterion at convergence: -14.4  Scaled residuals:   Min 1Q Median 3Q Max  -2.71477 -0.48471 0.03152 0.59618 2.34595   Random effects:  Groups Name Variance Std.Dev.  site_id (Intercept) 0.00000 0.0000   dx_group (Intercept) 0.01403 0.1184   Residual 0.03752 0.1937  Number of obs: 73, groups: site_id, 3; dx_group, 2  Fixed effects:  Estimate Std. Error df t value Pr(>\|t\|)  (Intercept) 0.229648 0.143183 5.184024 1.604 0.16758  AQ 0.009836 0.003188 50.951910 3.085 0.00329 ** sexmale 0.078615 0.090549 69.327959 0.868 0.38828  --- Signif. codes: 0 ‘***’ 0.001 ‘**’ 0.01 ‘*’ 0.05 ‘.’ 0.1 ‘ ’ 1  Correlation of Fixed Effects:  (Intr) AQ  AQ -0.532  sexmale -0.620 0.057 optimizer (nloptwrap) convergence code: 0 (OK) boundary (singular) fit: see help('isSingular') | # Fixed Effects  Parameter \| Coefficient \| 95% CI \| p ------------------------------------------------- (Intercept) \| 0.23 \| [-0.07, 0.52] \| 0.123 AQ \| 9.80e-03 \| [ 0.00, 0.02] \| 0.005 sexmale \| 0.08 \| [-0.10, 0.26] \| 0.397 |
